# Transcriptome analysis of barley (*Hordeum vulgare* L.) under waterlogging stress, and overexpression of the *HvADH4* gene confers waterlogging tolerance in transgenic *Arabidopsis*

**DOI:** 10.1186/s12870-023-04081-6

**Published:** 2023-01-30

**Authors:** Haiye Luan, Hongtao Li, Yu Li, Changyu Chen, Shufeng Li, Yu Wang, Ju Yang, Meng Xu, Huiquan Shen, Hailong Qiao, Jun Wang

**Affiliations:** 1 College of Marine and Biological Engineering, Yancheng Teachers University, Yancheng, 224002 Jiangsu China; 2 Jiangsu Provincial Key Laboratory of Coastal Wetland Bioresources and Environmental Protection, Yancheng, 224002 Jiangsu China; 3 Lianyungang Academy of Agricultural Sciences, Lianyungang, 222000 China; 4 Institute of Agricultural Science in Jiangsu Coastal Areas, Yancheng, 224002 China

**Keywords:** Barley, Waterlogging stress, Anatomical structure, Transcriptome analysis, ADH

## Abstract

**Background:**

Waterlogging is one of the major abiotic stresses in barley and greatly reduces grain yield and quality. To explore the mechanism controlling waterlogging tolerance in barley, physiological, anatomical and transcriptional analyses were performed in two contrasting barley varieties, viz. Franklin (susceptible) and TX9425 (tolerant).

**Results:**

Compared to Franklin, TX9425 had more adventitious roots and aerenchymas and higher antioxidant enzyme activities. A total of 3064 and 5693 differentially expressed genes (DEGs) were identified in TX9425 after 24 h and 72 h of waterlogging treatment, respectively, while 2297 and 8462 DEGs were identified in Franklin. The results suggested that TX9425 was less affected by waterlogging stress after 72 h of treatment. The DEGs were enriched mainly in energy metabolism, hormone regulation, reactive oxygen species (ROS) scavenging, and cell wall-modifying enzymes. Alcohol dehydrogenase (ADH) plays an important role in response to waterlogging stress. We found that *HvADH4* was significantly upregulated under waterlogging stress in TX9425. Transgenic *Arabidopsis* overexpressing *HvADH4* displayed higher activity of antioxidant enzymes and was more tolerant to waterlogging than the wild type (WT).

**Conclusions:**

The current results provide valuable information that will be of great value for the exploration of new candidate genes for molecular breeding of waterlogging tolerance in barley.

**Supplementary Information:**

The online version contains supplementary material available at 10.1186/s12870-023-04081-6.

## Background

Waterlogging stress, one of the major abiotic stresses affecting crop growth, has become more frequent, severe, and unpredictable due to the excessive water that also decreases the oxygen content in the soil and the nutrient absorption ability of the plant [1–3]. In general, barley is sensitive to waterlogging, which causes 40%-79% irreversible yield loss, depending on the genotype, growth stage and duration of waterlogging stress [4].

Root is the first organ responding to oxygen shortage, and it is critical for the maintenance of normal physiological activities in plants [5, 6]. As the one of the key features in waterlogging condition, the formation of adventitious roots exists widely in different plant species [7, 8]. New adventitious roots contain more aerenchymas, which can help maintain a hypoxia-tolerant pathway, store and exchange of gases within the different tissues, meanwhile reduce the number of oxygen-consuming cells [9]. This formation of aerenchyma requires ethylene, Ca^2+^ and reactive oxygen species (ROS) signaling [10, 11].

Antioxidant metabolism is one of the fundamental metabolic pathways under waterlogging stress [12]. The production of reactive oxygen species (ROS) is inevitable with plant’s exposure to waterlogging, this includes the superoxide radical (O_2_^.−^), hydroxyl radical (•OH) and hydrogen peroxide (H_2_O_2_), which readily attack leaf chloroplasts and ultimately lead to leaf chlorosis and senescence [13]. To avoid this, a series of antioxidant enzymes will be synthesized/activated to scavenge ROS, such as superoxide dismutase (SOD), peroxidase (POD) and catalase (CAT) [14]. The membrane structure and the activity of the cells would be destroyed by malondialdehyde (MDA), which has been utilized as a reliable indicator for waterlogging tolerance [15]. Thus, high levels of SOD, POD and CAT enzyme activity are critical for the survival of crop under waterlogging conditions [9, 15].

In addition, the energy metabolic pathway will be affected by oxygen deficiency. ATP is produced through glycolysis instead of oxidative phosphorylation [16]. Meanwhile, genes associated with ATP and carbohydrate catabolism, such as pyruvate decarboxylase 1 (PDC1), alcohol dehydrogenase 1 (ADH1) and sucrose phosphate synthase (SPS), were significantly upregulated in the process of anaerobic fermentation [17, 18]. The ADH genes of plants play an important role in the response to waterlogging. Some studies on the ADH function have been performed by transgenic assays, such as in *Arabidopsis* [19], soybeans [20], and kiwifruit [21]. Overexpression of the *GmAdh2* gene in transgenic soybean enhanced glycolysis and alcohol fermentation, and significantly increased the germination of transgenic lines under waterlogging [20]. Two genes in kiwifruit roots were also significantly induced after waterlogging treatment. The overexpression of *AdADH1* and *AdADH2* in kiwifruit enhanced waterlogging tolerance in transgenic *Arabidopsis* [21]. However, the function of ADH genes in response to waterlogging is different in various species.

Although numerous quantitative trait loci (QTL) studies have been conducted on waterlogging in barley, the genes responsible remain unidentified [22]. RNA-sequencing (RNA-seq) technology can identify key genes involved in various biological processes, and has been successfully used to reveal waterlogging responses in cucumber [23], wild soybean [24] and wheat [25]. In this study, physiological and dynamic RNA-seq analyses of on the roots of two barley cultivars exposed to waterlogging stress were conducted. The results obtained provide insights into the physiology and molecular mechanisms underlying the response of barley to waterlogging stress, which will facilitate barley genetic study and breeding applications.

## Results

### Phenotypic analysis of different barley varieties under waterlogging treatment

**Fig. 1:**
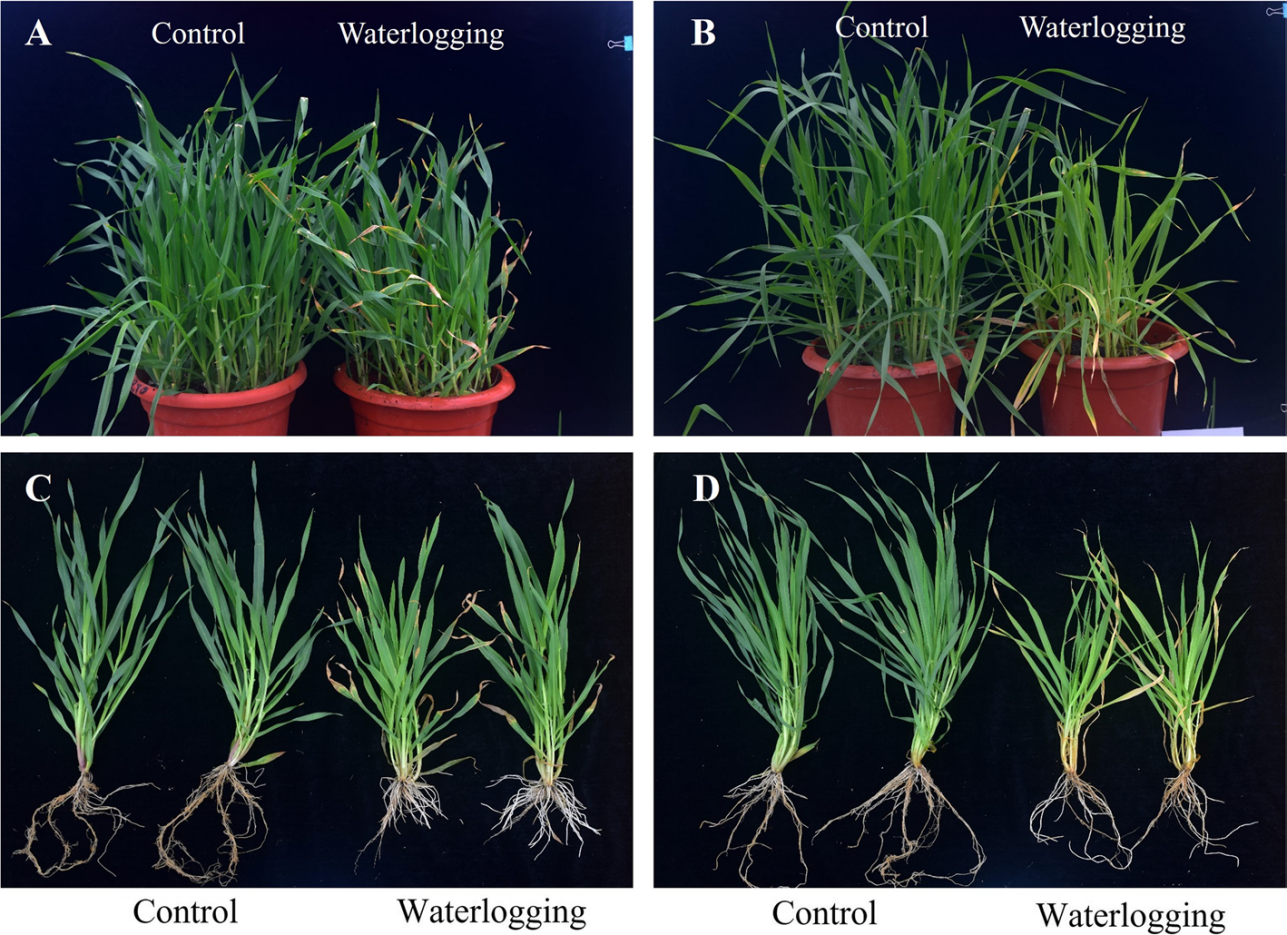
Morphological traits between waterlogging-sensitive Franklin and waterlogging-tolerant TX9425. (**A**) (**C**) TX9425; (**B**) (**D**) Franklin

The phenotypes of the two genotypes (TX9425 and Franklin) after 21 days of waterlogging treatment are shown in Fig. 1. No significant difference between Franklin and TX9425 under control conditions was observed in adventitious root parameters. However, after three weeks of waterlogging treatment, the length, surface area, volume and number of adventitious root of TX9425 significantly increased and the fold change value ranged from 2.36 to 4.06 compared to the control, while there was no significant difference in Franklin except adventitious root number (Table 1). The adventitious root number of TX9425 increased 4.06 times and that of Franklin increased more than 2.4 times. The adventitious roots of TX9425 became more developed than Franklin roots under waterlogging stress. Franklin leaves became more wilted and chlorotic than TX9425 leaves under waterlogging treatment. The plant height, tiller number, leaf area, shoot fresh weight and dry weight of Franklin significantly decreased. Compared with a small decline, values were detected in TX9425 leaves (Table 2). Therefore, the performance of Franklin and TX9425 displayed significant differences after three weeks of waterlogging treatment.

**Table 1:** The effect of waterlogging on the adventitious roots between TX9425 and Franklin

Treatment	Total Adventitious Length	Total Adventitious Surface Area	Adventitious Average Diameter	Total Adventitious Root Volume	Adventitious Root Number
	(cm)	(cm^2^)	(mm)	(cm^3^)	
Franklin					
Control	63.42 ± 12.36 a	14.8 ± 5.72a	0.75 ± 0.21a	0.29 ± 0.16a	6.17 ± 1.33a
Waterlogging	68.37 ± 12.31a	14.29 ± 3.04a	0.66 ± 0.04a	0.24 ± 0.06a	14.83 ± 2.33b
TX9425					
Control	68.41 ± 12.07a	13.93 ± 2.96a	0.62 ± 0.09a	0.22 ± 0.07a	5.83 ± 1.33a
Waterlogging	168.85 ± 13.87b	33.24 ± 8.38b	0.63 ± 0.03a	0.52 ± 0.13b	23.67 ± 3.83b

The different letters within a column for the same line represent significant difference between waterlogging treatment and control

**Table 2:** The effect of waterlogging on agronomic traits between TX9425 and Franklin

Treatment	Leaf Age	Leaf Chlorosis	Plant Height	Tillers	Leaf Area (cm^2^)	Shoot Fresh	Shoot Dry
			(cm)			Weight (g)	Weight (g)
Franklin							
Control	7.84 ± 0.44a	0.36 ± 0.45a	47.35 ± 2.39a	9.14 ± 3.25a	33.31 ± 4.32a	24 ± 1.13a	2.32 ± 0.15a
Waterlogging	7.97 ± 0.51a	4.96 ± 0.89b	28.38 ± 2.34b	6.75 ± 1.95b	16.67 ± 3.14b	9.97 ± 0.52b	1.35 ± 0.12b
TX9425							
Control	7.67 ± 0.4a	0.64 ± 0.49a	52.05 ± 3.48a	6.66 ± 1.54a	41.19 ± 6.15a	23.99 ± 1.4a	2.4 ± 0.2a
Waterlogging	7.86 ± 0.35a	1 ± 0.41b	45.11 ± 3.67b	6.41 ± 1.86a	34.15 ± 7.82b	20.24 ± 1.39b	2.3 ± 0.2a

The different letters within a column for the same line represent significant difference between waterlogging treatment and control

### Physiological and anatomical analysis of different barley varieties under waterlogging treatment

**Fig. 2:**
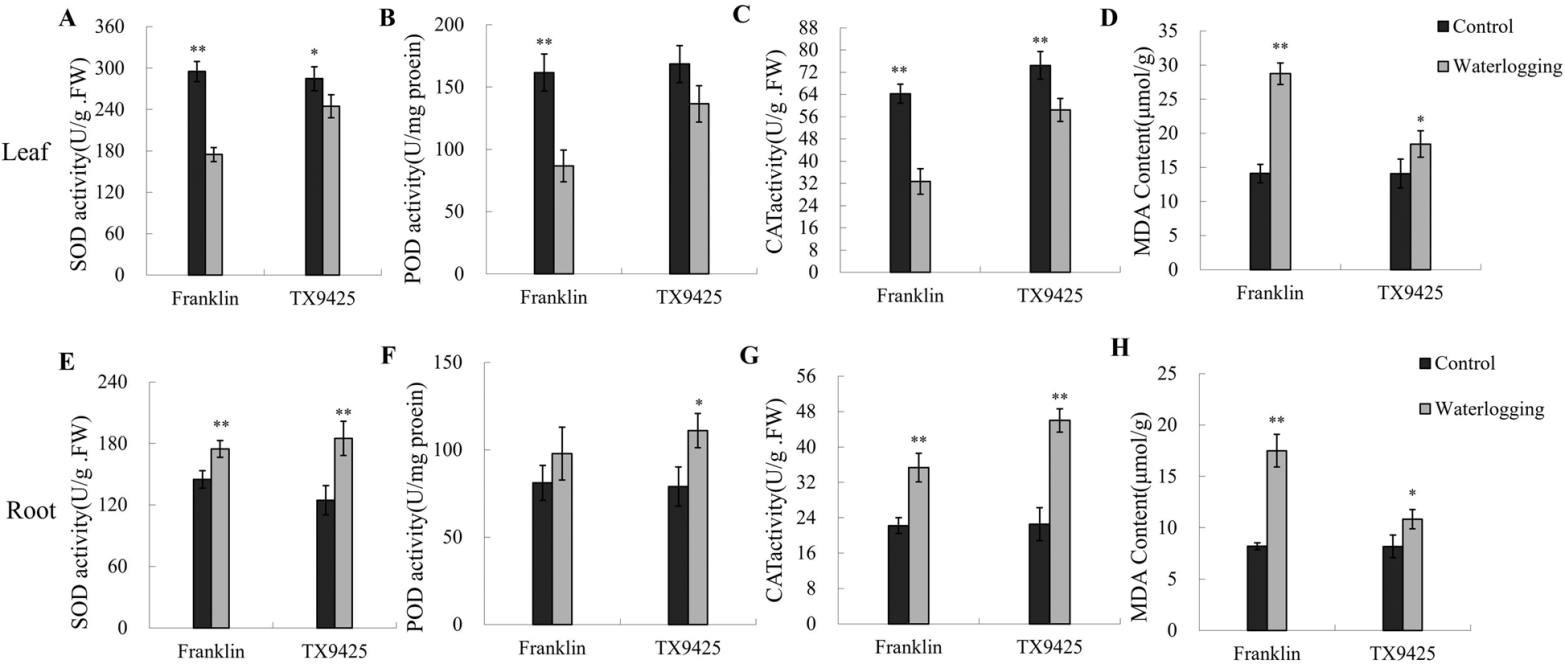
Effect of antioxidant enzymes activity and MDA content in leaf and root between TX9425 and Franklin. (**A**) SOD activity in leaf. (**B**) POD activity in leaf. (**C**) CAT activity in leaf. (**D**) MDA content in leaf. (**E**) SOD activity in root. (**F**) POD activity in root. (**G**) CAT activity in root. (**H**) MDA content in root. Results are the mean ± SD. *and** represent the significant differences at *p* < *0.05* and *p* < *0.01*, respectively

As shown in Fig. 2 A-C, a significant genotype difference in the activities of SOD, CAT and POD in leaves was found. The antioxidant enzyme activity in both varieties decreased under waterlogging, while the decrease in the tolerance of TX9425 was lower. Under waterlogging treatment, the increase in MDA content of Franklin was approximately 2.1-fold, but the MDA content of TX9425 increased by only 1.3-fold (Fig. 2D). The root SOD activity of Franklin and TX9425 increased by 1.2- and 1.5-fold, respectively (Fig. 2E). The CAT activity of Franklin roots increased by 1.6-fold, and the CAT activity of TX9425 increased by 2.1-fold (Fig. 2G). Moreover, the POD enzyme activity of TX9425 increased by 1.4-fold, and no significant difference was observed in the roots of Franklin between the waterlogging treatment and the control (Fig. 2F). In contrast, the MDA content of Franklin increased by 2.1-fold compared with the MDA content of the control in roots, but the change in TX9425 was not significant (Fig. 2H). Therefore, TX9425 showed higher activity of antioxidant enzymes in leaves and roots after waterlogging, suggesting that TX9425 suffered less membrane damage than Franklin.

**Fig. 3:**
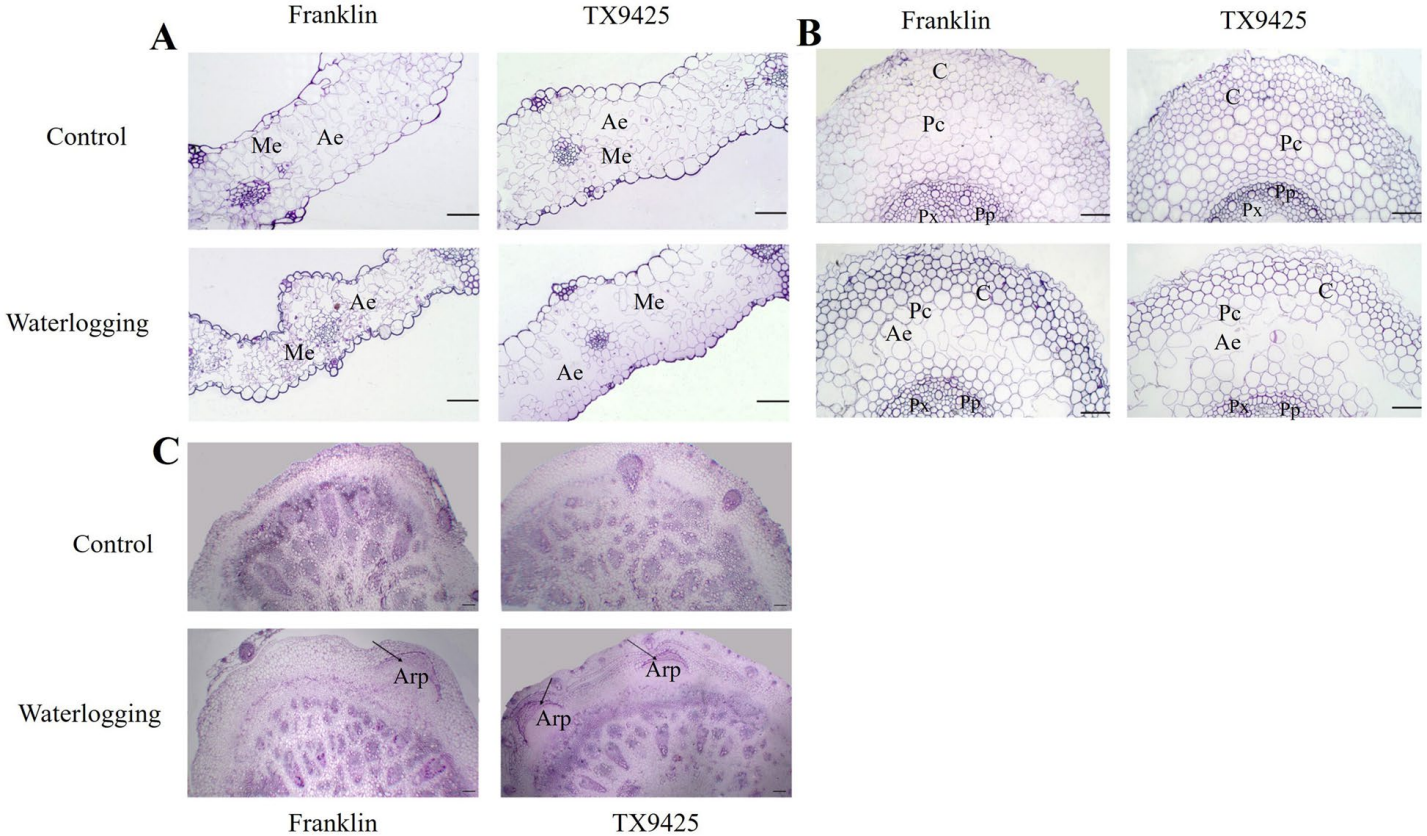
Transverse of leaf, adventitious root and root nodal between waterlogging-sensitive Franklin and waterlogging-tolerant TX9425. **A** Transverse of leaf in two genotypes; (**B**) Transverse of adventitious root in two genotypes; (**C**) Transverse of root nodal in two genotypes. Note: (**A**) Me, mesophyll cells; Ep, epidermis cell; Ae, aerenchyma. (**B**) C, Cortex; Pc, Parenchyma Cell; Pp, Primary Phloem; Px, Primary Xylem; Ae, Aerenchyma. **C** Arp, adventitious root primordia. Scale bar: 50 μm

Barley leaf anatomy is a typical monocotyledonous type consisting of epidermis, mesophyll and vascular tissue. Intercellular spaces existed among the mesophyll cells in the control. Under waterlogging, mesophyll cells of Franklin were severely damaged; in contrast, the leaves of TX9425 developed more lysigenous aerenchyma under waterlogging compared with the control (Fig. 3A). The adventitious root of barley was composed of the epidermis, cortex and cylinder of vascular tissues. Cortex parenchyma cells of adventitious roots formed a larger number of lysigenous aerenchyma under waterlogging conditions, compared with small intercellular space under control conditions. Remarkably, the proportion of TX9425 aerenchyma was significantly higher than Franklin after three weeks of treatment (Fig. 3B). Under waterlogging, adventitious roots were formed in the section of the shoot base in both lines, and more adventitious root primordia were observed in TX9425 than Franklin. Otherwise, in the absence of waterlogging, few adventitious roots were found in either accession (Fig. 3C, Table 2).

### Analysis of barley root transcriptome under waterlogging stress

**Fig. 4:**
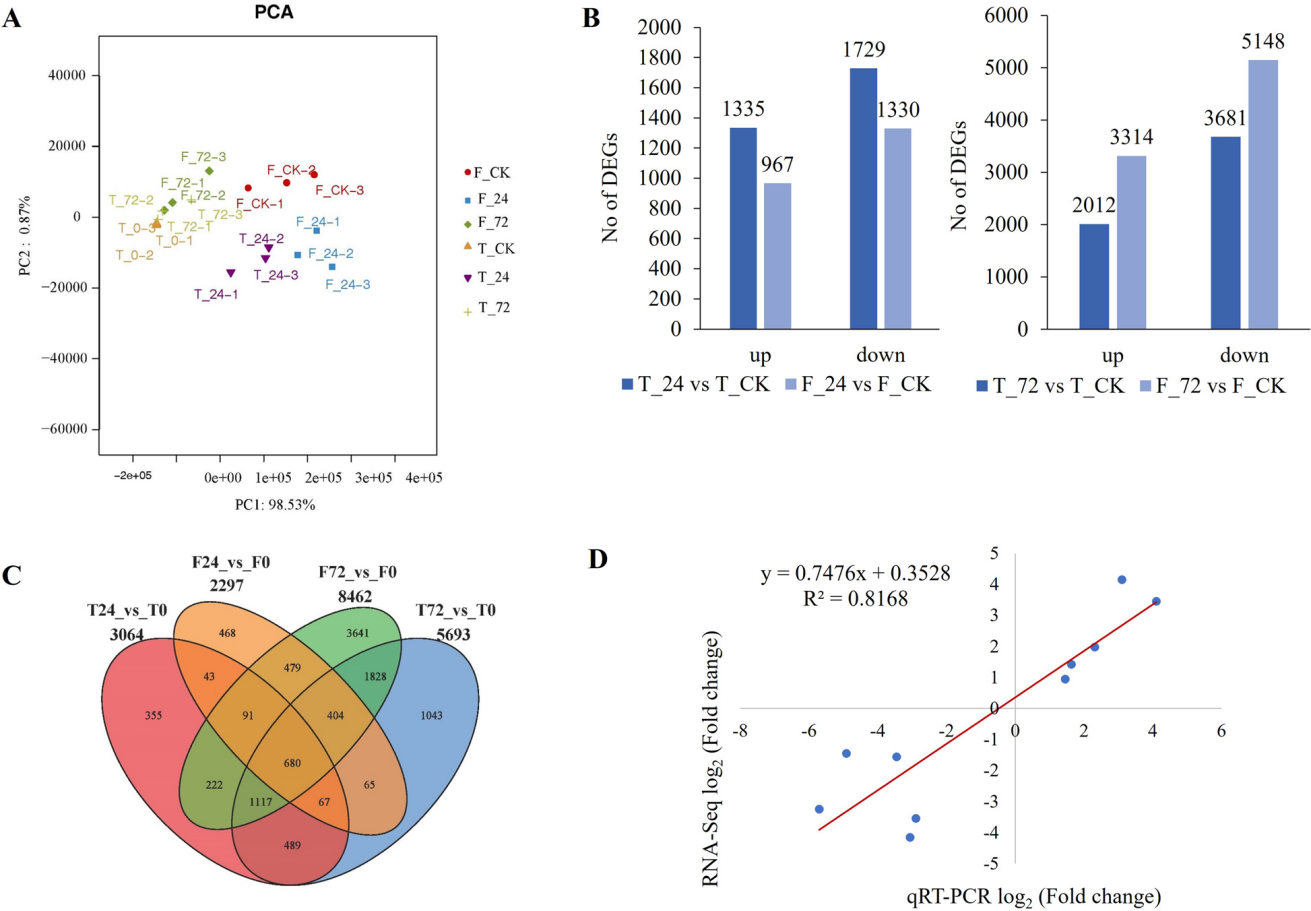
Transcriptome analysis in roots of TX9425 and Franklin under control and waterlogging conditions. (**A**) Principal component analysis (PCA) of transcript changes separates the samples under control and waterlogging (24 h and 72 h waterlogging treatment) conditions. (**B**) Venn diagram depicting the number of all DEGs expressed in root tissues of TX9425 and Franklin genotypes under stress and control conditions. (**C**) The correlation between the log_2_ (fold change) of 10 DEGs in the qRT-PCR experiment (x axis) and in the RNA-seq experiments (y axis). Gene relative expression was calculated by 2^−△△Ct^ method. *Actin* was used as the reference gene. Data used were means of three replicates

To reveal the molecular mechanisms of barley in response to waterlogging stress, roots were collected from TX9425 and Franklin after 0 h, 24 h and 72 h waterlogging treatments. Each sample was subjected to three replicate treatments, and a total of 18 libraries were constructed. A high-throughput Illumina sequencing platform was used to sequence the transcriptome of barley. After removing adaptor sequences, low-quality reads, and reads with more than 10% ambiguous “N” bases, 2.87–7.58 GB data were obtained from each sample. The Q20 values of all transcriptomes were all above 96.42%, and the Q30 values were at least 92.41%, indicating high-quality sequencing data in the RNA-seq experiments (Table S1). On average, more than 63% of the valid reads were mapped into the reference barley genome. Principal component analysis (PCA) was conducted on the RNA-seq dataset of 18 samples. The control and treatment samples of the two genotypes were clearly separated by the first principal component (PC1), which accounted for 98.53% of the total variation (Fig. 4A).

### Identification of DEGs in two barley varieties in response to waterlogging stress

We further compared the DEGs in the two barley varieties subjected to waterlogging stress. We found a total of 3064 DEGs in TX9425 and 2297 DEGs in Franklin after 24 h of waterlogging stress compared to the control, by using the parameters of log_2_ fold change ≥ 1 and *P* value ≤ 0.05. A total of 1335 DEGs were upregulated and 1729 DEGs were downregulated in TX9425, while there were 967 upregulated genes and 1330 downregulated genes in Franklin (Fig. 4B). By comparing the transcriptome profiles of TX9425 and Franklin, a total of 2183 DEGs were observed to be uniquely expressed in TX9425 only, whereas 1416 DEGs were distinctively found in Franklin under the 24 h waterlogging treatment. In addition, 881 DEGs were common between the two genotypes (Fig. 4C).

A total of 5693 DEGs and 8462 DEGs were identified under waterlogging treatment (72 h) vs the control in TX9425 and Franklin, respectively. A total of 2012 DEGs were upregulated and 3681 DEGs were downregulated in TX9425, while there were 3314 upregulated genes and 5148 downregulated genes in Franklin. There were more DEGs after 72 h of waterlogging stress than after 24 h of waterlogging stress. The number of DEGs was significantly different between TX9425 and Franklin (Fig. 4B). A total of 1664 DEGs were uniquely expressed in TX9425 only, whereas a total of 4083 DEGs were distinctively found in Franklin under 72 h of waterlogging treatment. In addition, 4029 DEGs were common between the two genotypes (Fig. 4C).

In addition, to verify the reliability of the RNA-seq data, 10 DEGs were randomly selected for qRT-PCR analysis. Significantly positive correlations were observed between qRT-PCR and RNA sequencing data (r^2^ = 0.82). These results suggested that the RNA-seq data were credible (Fig. 4D).

### Functional annotation of waterlogging-responsive DEGs

**Fig. 5:**
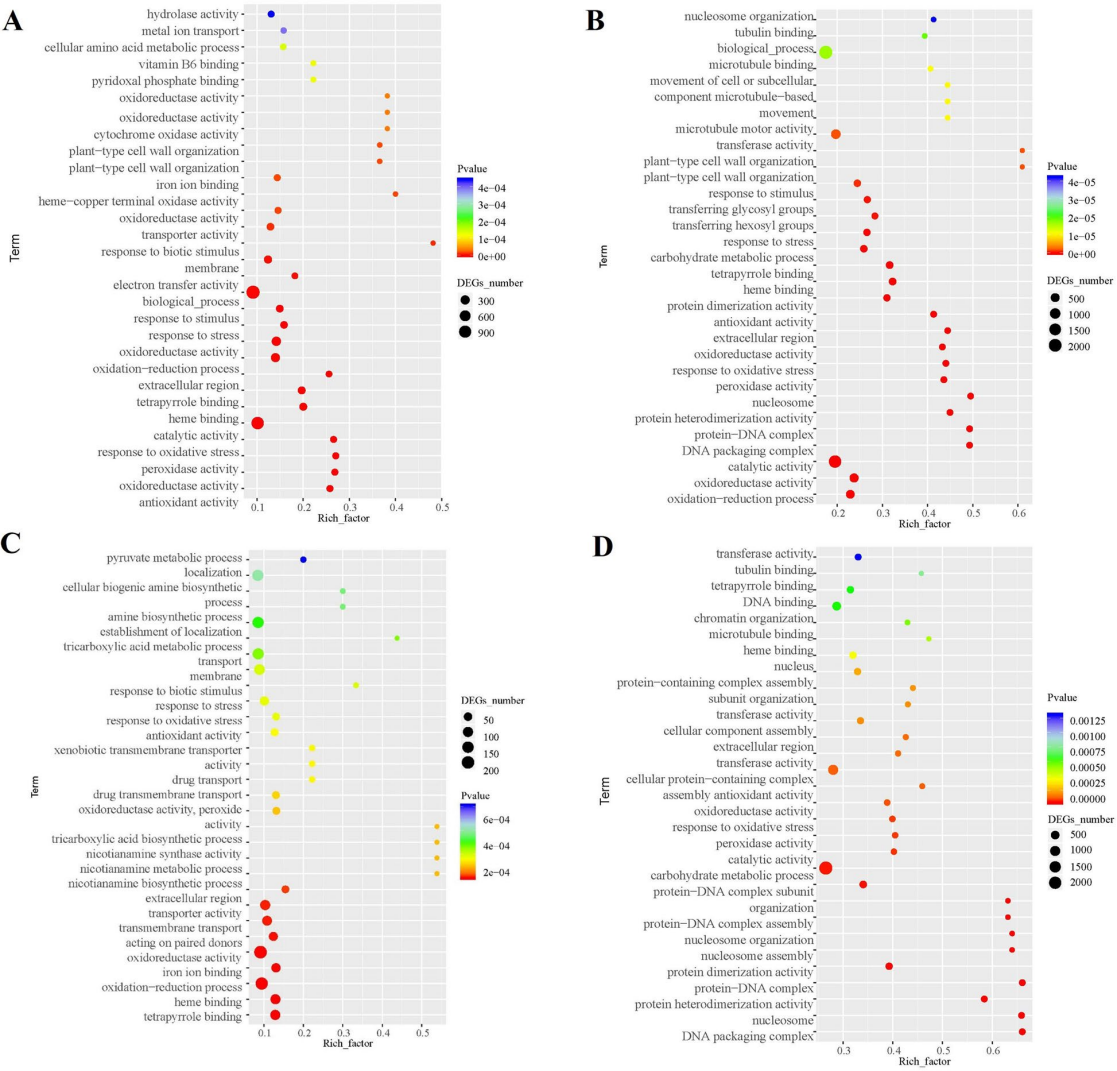
Gene ontology (GO) enrichment analysis of differentially expressed genes (DEGs) in roots of TX9425 and Franklin under waterlogging stress. (**A**) GO enrichment analysis of TX9425 at 24 h waterlogging stress. (**B**) GO enrichment analysis of TX9425 at 72 h waterlogging stress. (**C**) GO enrichment analysis of Franklin at 24 h waterlogging stress. (**D**) GO enrichment analysis of Franklin at 72 h waterlogging stress

Gene Ontology (GO) functional classification analysis was performed to categorize the functions of DEGs during waterlogging stress (Table S2). As determined through a GO enrichment analysis of these DEGs, the DEGs in TX9425 and Franklin under 24 h waterlogging stress functioned mostly in biological processes, metabolic processes, transferase activity and catalytic activity (Fig. 5 A, B). After 72 h of waterlogging, the DEGs of TX9425 functioned mainly in metabolic processes, biological processes, organic cyclic compound binding, heterocyclic compound binding and catalytic activity. However, the DEGs in Franklin mostly showed localization, oxidation–reduction process, protein binding and catalytic activity (Fig. 5 C, D, Table S2).

For KEGG pathway enrichment analysis, these DEGs were significantly (*p* < 0.01) enriched into 27 KEGG pathways (Table S3). Under 24 h of waterlogging stress, the DEGs of TX9425 were enriched mostly in metabolic pathways and biosynthesis of secondary metabolites. However, the DEGs in Franklin were associated primarily with biosynthesis of secondary metabolites and phenylpropanoid biosynthesis. Under 72 h of waterlogging stress, the DEGs of TX9425 were enriched mostly in the biosynthesis of secondary metabolites, MAPK signalling pathway, toll-like receptor signalling pathway. However, the DEGs in Franklin were associated primarily with biosynthesis of secondary metabolites, biosynthesis of antibiotics and phenylpropanoid biosynthesis.

### Analysis of DEGs related to energy metabolism, hormone regulation, ROS scavenging and cell wall modifying enzymes

Energy deprivation is one of the major factors affecting survival of waterlogged plants. The KEGG enrichment analysis showed that many DEGs were involved in starch and sucrose metabolism and the glycolysis/fermentation pathway. As expected, we found that several DEGs, such as sucrose synthase, pyruvate kinase family protein, ATP-dependent 6-phosphofructokinase, alpha-amylase/subtilisin inhibitor, and fructose-bisphosphate aldolase 2, were significantly accumulated in both TX9425 and Franklin.

In addition, some DEGs involved in the glycolysis/fermentation pathway such as alanine aminotransferase, glyceraldehyde-3-phosphate dehydrogenase C2, alcohol dehydrogenase 1, L-lactate dehydrogenase A, and pyruvate decarboxylase-2, were also significantly induced by waterlogging stress in the two genotypes. In this study, we found that some genes had different expression levels in the two varieties. For example, the pyruvate kinase family protein *(HORVU2Hr1G040570)* and fructose-bisphosphate aldolase 2 *(HORVU3Hr1G088500)* were induced at higher levels in TX9425 than in Franklin after 24 or 72 h of waterlogging treatment. The expression levels of ATP-dependent 6-phosphofructokinase *(HORVU5Hr1G019030)*, alpha-amylase/trypsin inhibitor *(HORVU7Hr1G035020)*, and alcohol dehydrogenase 1* (HORVU1Hr1G082250*, *HORVU4Hr1G016810)* first increased and then decreased in TX9425, while they continuously increased in Franklin. Consequently, TX9425 had a greater energy state than Franklin under waterlogging stress (Table 3).

**Table 3:** Selected differentially expressed genes with altered expression in roots of waterlogged TX9425 and Franklin that are involved in major metabolism pathways

Geneid	Gene description	FPKM					
		F-CK	F-24	F-72	T-CK	T-24	T-72
**Starch and sucrose metabolism**							
HORVU7Hr1G007220	sucrose synthase 1	2.19	1.41	0.27	9.91	3.13	1.70
HORVU7Hr1G033230	sucrose synthase 4	170.53	595.48	1873.62	191.26	1156.87	1429.41
HORVU1Hr1G054380	Pyruvate kinase family protein	41.59	31.25	2.48	55.72	14.21	7.39
HORVU2Hr1G040570	Pyruvate kinase family protein	11.24	36.70	60.56	18.77	168.36	174.52
HORVU2Hr1G119460	Pyruvate kinase family protein	2.23	6.65	15.33	2.91	36.25	44.43
HORVU5Hr1G041120	Pyruvate kinase family protein	60.85	137.65	328.22	64.00	302.60	461.59
HORVU0Hr1G005720	Pyruvate kinase family protein	5.60	12.88	34.47	16.63	50.12	77.94
HORVU1Hr1G080480	6-phosphogluconate dehydrogenase, decarboxylating 1	28.03	26.04	7.75	79.08	58.81	10.22
HORVU1Hr1G040620	Triosephosphate isomerase	1.15	2.60	38.82	2.56	3.95	43.03
HORVU6Hr1G070270	ATP-dependent 6-phosphofructokinase 7	81.36	68.75	9.66	121.88	23.88	21.72
HORVU7Hr1G022500	ATP-dependent 6-phosphofructokinase 3	27.42	7.60	5.75	24.62	6.79	4.27
HORVU1Hr1G075550	ATP-dependent 6-phosphofructokinase 3	19.59	241.12	618.01	38.70	408.91	499.23
HORVU3Hr1G019580	ATP-dependent 6-phosphofructokinase 3	27.63	55.14	265.15	78.59	104.78	366.03
HORVU5Hr1G019030	ATP-dependent 6-phosphofructokinase	18.25	122.03	219.02	38.73	305.77	224.04
HORVU2Hr1G090750	Alpha-amylase/subtilisin inhibitor	0.25	24.08	338.12	2.01	27.35	202.64
HORVU7Hr1G035020	Alpha-amylase/trypsin inhibitor	0.25	1.55	1.76	0.37	4.54	2.17
HORVU7Hr1G091250	alpha-amylase-like	2.12	24.51	19.34	2.19	46.98	13.53
HORVU7Hr1G115590	sucrose phosphate synthase 1F	13.69	20.97	66.17	14.33	55.22	56.63
HORVU7Hr1G000250	Acid beta-fructofuranosidase	6.13	0.56	0.61	2.84	0.23	0.44
HORVU7Hr1G001070	Acid beta-fructofuranosidase	5.13	1.72	3.87	11.30	1.55	2.24
HORVU1Hr1G056180	D-3-phosphoglycerate dehydrogenase	8.39	2.09	0.37	8.00	1.64	0.79
HORVU3Hr1G088500	fructose-bisphosphate aldolase 2	12.34	27.93	121.48	19.82	108.27	268.93
HORVU3Hr1G088540	fructose-bisphosphate aldolase 2	44.59	89.50	526.85	146.54	225.28	545.16
**Glycolysis Fermentation**							
HORVU1Hr1G018540	alanine aminotransferase 2	38.03	219.48	875.01	64.50	522.47	581.12
HORVU7Hr1G074230	alanine aminotransferase 2	0.22	1.25	9.52	0.98	5.92	4.70
HORVU7Hr1G074250	alanine aminotransferase 2	6.19	30.15	137.90	9.43	78.77	84.65
HORVU7Hr1G108580	glyceraldehyde-3-phosphate dehydrogenase C2	21.34	14.19	1.19	39.88	5.56	4.67
HORVU6Hr1G054520	glyceraldehyde-3-phosphate dehydrogenase C2	560.85	1572.08	4867.10	1025.33	2136.81	5577.50
HORVU2Hr1G036110	NADP-dependent glyceraldehyde-3-phosphate dehydrogenase	0.36	6.29	23.60	0.95	11.21	15.29
HORVU1Hr1G010130	alcohol dehydrogenase 1	18.99	2.80	1.32	17.74	3.45	2.35
HORVU2Hr1G010010	alcohol dehydrogenase 1	8.95	2.76	0.38	7.57	1.63	1.04
HORVU2Hr1G068010	Alcohol dehydrogenase	9.06	2.53	1.62	8.74	4.77	1.15
HORVU4Hr1G078470	alcohol dehydrogenase 1	0.55	0.21	0.12	1.75	0.21	0.25
HORVU1Hr1G003130	alcohol dehydrogenase 1	0.53	3.20	3.72	1.29	5.59	7.40
HORVU1Hr1G082250	alcohol dehydrogenase 1	34.07	416.15	459.58	30.92	1542.59	782.58
HORVU2Hr1G020900	alcohol dehydrogenase 1	1.22	9.36	11.36	3.39	26.86	15.62
HORVU3Hr1G034820	alcohol dehydrogenase 1	5.99	45.60	59.31	17.05	106.98	89.35
HORVU4Hr1G016770	alcohol dehydrogenase 1	29.40	374.38	571.97	51.95	747.24	590.62
HORVU4Hr1G016780	alcohol dehydrogenase 1	6.86	48.03	113.90	9.76	153.13	146.48
HORVU4Hr1G016810	alcohol dehydrogenase 1	18.08	143.60	165.32	18.92	307.64	232.83
HORVU5Hr1G010490	alcohol dehydrogenase 1	1.51	7.43	18.74	3.26	20.16	19.92
HORVU5Hr1G051820	alcohol dehydrogenase 1	0.62	8.42	8.08	3.04	17.87	15.35
HORVU6Hr1G063060	alcohol dehydrogenase 1	0.41	1.38	0.91	0.20	2.13	1.73
HORVU7Hr1G058160	alcohol dehydrogenase 1	4.95	32.72	52.35	15.49	90.89	74.97
HORVU0Hr1G008870	alcohol dehydrogenase 1	9.23	66.82	89.40	27.74	174.75	126.64
HORVU0Hr1G010220	alcohol dehydrogenase 1	0.56	4.80	8.12	2.21	13.53	10.68
HORVU2Hr1G012830	L-lactate dehydrogenase B	1.21	2.35	34.24	4.59	5.18	76.68
HORVU6Hr1G015500	L-lactate dehydrogenase A	13.25	58.67	142.00	25.75	146.50	219.90
HORVU7Hr1G096800	L-lactate dehydrogenase A	0.73	17.89	88.39	7.63	31.71	147.17
HORVU4Hr1G056050	pyruvate decarboxylase-2	48.68	416.00	262.83	57.85	749.91	440.42
**Hormones-related genes**							
HORVU1Hr1G020410	1-aminocyclopropane-1-carboxylate oxidase	3.98	2.51	0.20	16.36	1.55	0.78
HORVU1Hr1G020450	1-aminocyclopropane-1-carboxylate oxidase	38.30	7.83	3.66	87.89	9.04	9.17
HORVU2Hr1G094230	1-aminocyclopropane-1-carboxylate synthase 11	4.46	3.53	2.89	34.77	3.42	6.86
HORVU4Hr1G009800	1-aminocyclopropane-1-carboxylate synthase 11	1.35	4.45	0.50	24.33	12.33	2.66
HORVU4Hr1G017660	1-aminocyclopropane-1-carboxylate oxidase	4.88	0.96	0.00	5.02	0.49	0.34
HORVU7Hr1G086650	1-aminocyclopropane-1-carboxylate oxidase 1	1.65	0.45	0.03	1.87	1.20	0.13
HORVU5Hr1G067490	1-aminocyclopropane-1-carboxylate oxidase 1	0.66	8.82	7.23	1.01	22.42	14.74
HORVU5Hr1G067530	1-aminocyclopropane-1-carboxylate oxidase 1	0.74	2.04	2.44	0.59	6.02	6.02
HORVU1Hr1G051780	auxin response factor 4	1.09	0.23	0.00	2.02	0.33	0.10
HORVU1Hr1G076690	Auxin response factor 14	0.37	0.34	0.07	3.09	0.26	0.21
HORVU2Hr1G022640	Auxin-induced protein-like	6.51	1.18	0.00	8.83	0.71	0.00
HORVU2Hr1G092070	SAUR-like auxin-responsive protein family	5.25	2.61	0.62	5.94	5.66	1.04
HORVU2Hr1G100360	SAUR-like auxin-responsive protein family	3.44	1.06	0.00	6.69	0.33	0.13
HORVU2Hr1G122970	Auxin-induced protein 5NG4	0.93	0.09	0.54	3.96	0.27	0.22
HORVU3Hr1G009330	auxin response factor 19	2.27	0.40	0.24	6.87	0.69	0.19
HORVU3Hr1G072340	Auxin response factor 3	4.09	1.45	0.41	5.70	1.19	1.18
HORVU3Hr1G080640	Auxin efflux carrier family protein	18.61	9.54	3.89	30.69	13.84	6.69
HORVU4Hr1G021650	auxin response factor 17	0.91	0.14	0.07	1.47	0.22	0.23
HORVU5Hr1G076690	SAUR-like auxin-responsive protein family	1.73	0.72	0.16	2.47	1.38	0.03
HORVU5Hr1G076740	SAUR-like auxin-responsive protein family	4.47	2.85	0.83	12.74	10.82	1.13
HORVU5Hr1G094220	Auxin-responsive protein IAA13	93.35	46.33	5.09	139.87	26.69	15.81
HORVU5Hr1G094270	Auxin-responsive protein IAA13	21.81	10.25	0.96	32.76	6.35	2.25
HORVU6Hr1G031510	Auxin transporter-like protein 3	2.09	0.63	0.00	5.19	0.54	0.29
HORVU7Hr1G033820	auxin response factor 19	5.33	3.18	1.87	28.70	3.38	2.95
HORVU7Hr1G077110	Auxin-responsive protein IAA25	1.12	0.77	0.18	2.20	0.95	0.34
HORVU7Hr1G084940	Auxin-responsive protein IAA23	4.48	2.90	0.19	7.29	4.80	1.07
HORVU7Hr1G110470	Auxin efflux carrier family protein	10.86	5.29	0.36	14.77	3.75	0.86
HORVU1Hr1G025670	Auxin-responsive protein IAA15	16.27	8.04	24.12	18.81	16.03	52.17
HORVU3Hr1G022540	Auxin-responsive protein IAA1	14.85	12.26	47.68	29.98	26.61	83.85
HORVU3Hr1G064590	auxin response factor 20	0.00	0.00	0.75	0.04	0.09	5.78
HORVU3Hr1G078620	Auxin efflux carrier family protein	4.52	28.60	25.68	25.68	108.53	87.38
HORVU3Hr1G084840	Auxin response factor	0.21	19.44	208.99	0.29	14.53	436.04
HORVU4Hr1G002550	SAUR-like auxin-responsive protein family	0.15	0.51	0.31	0.53	0.00	2.68
HORVU4Hr1G026680	Auxin efflux carrier family protein	0.51	0.07	0.02	0.54	0.43	1.47
HORVU5Hr1G044470	Auxin-induced protein 5NG4	0.14	1.10	6.73	0.49	2.38	5.85
HORVU5Hr1G062580	SAUR-like auxin-responsive protein family	8.56	17.50	65.80	14.30	18.40	36.89
HORVU6Hr1G091230	Auxin-binding protein 1	0.55	0.67	1.07	1.30	4.33	9.51
HORVU7Hr1G096870	SAUR-like auxin-responsive protein family	13.44	9.93	32.84	10.64	15.11	32.34
HORVU7Hr1G107370	SAUR-like auxin-responsive protein family	0.57	0.25	9.24	1.01	0.23	12.97
**ROS scavengers**							
HORVU1Hr1G021150	Glutathione S-transferase family protein	20.29	14.97	2.91	47.13	21.61	5.54
HORVU1Hr1G049120	Glutathione S-transferase family protein	40.06	36.45	10.00	45.58	36.60	7.52
HORVU1Hr1G049190	Glutathione S-transferase family protein	7.78	13.82	0.24	47.58	21.55	1.52
HORVU1Hr1G049250	Glutathione S-transferase family protein	5.97	1.45	0.00	7.56	0.43	0.42
HORVU1Hr1G052470	Glutathione S-transferase family protein	6.34	5.44	1.99	24.71	3.94	5.17
HORVU2Hr1G095460	Glutathione S-transferase family protein	77.53	25.08	15.77	153.62	55.61	24.25
HORVU2Hr1G124300	Glutathione S-transferase family protein	2.13	0.63	0.90	5.12	0.85	0.27
HORVU2Hr1G124310	Glutathione S-transferase family protein	11.84	14.65	2.45	10.70	6.47	1.02
HORVU2Hr1G124330	Glutathione S-transferase family protein	0.94	0.41	0.32	6.81	2.21	0.51
HORVU3Hr1G083520	Glutathione S-transferase family protein	2.97	1.23	0.00	4.31	0.32	0.09
HORVU3Hr1G098820	Glutathione S-transferase family protein	0.77	1.63	1.97	4.65	1.93	0.92
HORVU3Hr1G106450	Glutathione S-transferase family protein	5.42	3.32	0.03	12.25	3.49	1.46
HORVU3Hr1G107160	Glutathione S-transferase family protein	2.80	1.03	0.32	1.24	0.28	0.18
HORVU3Hr1G107170	Glutathione S-transferase family protein	17.33	4.15	1.28	13.53	5.13	1.25
HORVU3Hr1G107280	Glutathione S-transferase family protein	22.65	4.01	0.92	19.76	5.15	3.52
HORVU3Hr1G111150	Glutathione S-transferase family protein	8.49	3.00	0.17	7.35	2.26	0.50
HORVU4Hr1G057910	Glutathione S-transferase family protein	45.79	62.37	1.36	69.46	19.80	7.04
HORVU5Hr1G006330	Glutathione S-transferase family protein	33.09	17.62	3.17	109.85	31.20	15.66
HORVU5Hr1G006630	Glutathione S-transferase family protein	4.93	3.29	0.63	16.19	2.88	1.87
HORVU5Hr1G104670	Glutathione S-transferase family protein	0.24	0.60	0.19	8.14	0.96	0.41
HORVU6Hr1G011120	Glutathione S-transferase family protein	40.94	16.81	4.76	120.50	22.45	26.38
HORVU6Hr1G026810	Glutathione S-transferase family protein	7.22	30.96	0.72	45.66	14.92	4.34
HORVU7Hr1G008830	Glutathione S-transferase family protein	3.47	0.84	0.06	7.61	1.76	0.44
HORVU7Hr1G108570	Glutathione S-transferase family protein	13.32	7.11	0.04	30.90	4.35	1.32
HORVU1Hr1G001560	Glutathione S-transferase family protein	2.65	3.41	2.31	2.82	14.22	8.07
HORVU1Hr1G002160	Glutathione S-transferase family protein	1.27	3.36	5.76	1.40	7.25	17.19
HORVU3Hr1G010480	Glutathione S-transferase family protein	4.78	2.50	11.67	2.13	2.72	5.19
HORVU3Hr1G107350	Glutathione S-transferase family protein	0.20	0.55	0.31	0.11	2.95	1.07
HORVU3Hr1G117370	Glutathione S-transferase family protein	2.00	3.13	5.54	4.94	23.28	17.21
HORVU3Hr1G117390	Glutathione S-transferase family protein	1.67	6.32	6.78	14.79	71.12	53.67
HORVU4Hr1G082810	Glutathione S-transferase family protein	0.18	0.08	2.75	0.62	0.29	3.83
HORVU5Hr1G103420	Glutathione S-transferase family protein	1.89	39.82	89.04	4.48	52.10	109.96
HORVU1Hr1G075760	Peroxidase 2	3.98	1.95	0.07	10.97	1.27	1.61
HORVU1Hr1G075780	Peroxidase 2	10.57	3.44	0.36	22.40	2.01	4.40
HORVU3Hr1G083190	Peroxidase 2	13.08	5.58	1.79	39.99	7.52	2.37
HORVU1Hr1G016720	Peroxidase superfamily protein	2.62	0.93	0.11	1.96	0.77	0.17
HORVU1Hr1G016770	Peroxidase superfamily protein	19.32	2.22	3.63	13.08	1.05	0.11
HORVU1Hr1G016820	Peroxidase superfamily protein	12.96	3.75	0.76	29.93	2.63	0.77
HORVU1Hr1G016840	Peroxidase superfamily protein	20.16	10.76	1.44	101.53	19.32	7.88
HORVU1Hr1G016870	Peroxidase superfamily protein	4.30	2.85	0.23	16.93	1.85	0.49
HORVU1Hr1G051740	Peroxidase superfamily protein	23.96	4.68	0.76	26.70	2.86	3.21
HORVU1Hr1G054640	Peroxidase superfamily protein	4.29	2.51	0.15	9.40	1.70	0.33
HORVU1Hr1G066540	Peroxidase superfamily protein	10.31	2.82	0.00	22.85	2.21	0.00
HORVU1Hr1G066550	Peroxidase superfamily protein	4.03	2.63	0.17	27.35	2.75	0.13
HORVU1Hr1G066580	Peroxidase superfamily protein	21.22	4.97	0.00	33.40	2.95	0.00
HORVU1Hr1G066600	Peroxidase superfamily protein	9.20	1.62	0.00	14.85	1.40	0.00
HORVU1Hr1G066610	Peroxidase superfamily protein	13.84	2.17	0.00	26.05	2.95	0.22
HORVU1Hr1G069000	Peroxidase superfamily protein	10.67	1.55	0.00	13.09	1.10	0.00
HORVU1Hr1G075770	Peroxidase superfamily protein	1.46	1.61	0.00	4.62	0.27	0.23
HORVU2Hr1G018480	Peroxidase superfamily protein	7.25	1.42	0.30	2.77	0.67	0.67
HORVU2Hr1G018550	Peroxidase superfamily protein	3.93	0.10	0.00	4.22	0.31	0.00
HORVU2Hr1G025730	Peroxidase superfamily protein	1.13	0.39	0.00	3.76	0.14	0.00
HORVU2Hr1G025740	Peroxidase superfamily protein	1.91	0.08	0.00	3.34	0.17	0.00
HORVU2Hr1G026370	Peroxidase superfamily protein	35.29	8.81	0.21	26.28	2.83	4.77
HORVU2Hr1G026380	Peroxidase superfamily protein	1.02	0.35	0.00	3.75	0.31	0.00
HORVU2Hr1G026420	Peroxidase superfamily protein	9.87	1.99	0.00	13.33	1.17	0.00
HORVU2Hr1G026640	Peroxidase superfamily protein	9.45	1.09	0.00	12.25	1.17	0.06
HORVU2Hr1G044340	Peroxidase superfamily protein	9.25	1.09	0.54	0.56	0.18	0.00
HORVU2Hr1G064460	Peroxidase superfamily protein	6.56	1.81	0.05	10.18	2.78	0.52
HORVU2Hr1G074680	Peroxidase superfamily protein	58.60	14.15	2.15	56.74	11.20	3.81
HORVU2Hr1G107350	Peroxidase superfamily protein	2.45	0.60	0.08	2.44	0.30	0.31
HORVU2Hr1G124970	Peroxidase superfamily protein	148.81	30.69	7.55	100.53	31.47	14.85
HORVU2Hr1G124980	Peroxidase superfamily protein	18.98	7.16	0.29	26.13	4.81	1.61
HORVU2Hr1G125050	Peroxidase superfamily protein	29.43	3.99	0.00	29.13	4.32	0.00
HORVU2Hr1G125090	Peroxidase superfamily protein	167.61	17.59	0.20	114.24	18.69	0.74
HORVU3Hr1G027850	Peroxidase superfamily protein	19.55	6.03	0.23	15.89	3.58	2.07
HORVU3Hr1G036780	Peroxidase superfamily protein	9.12	4.44	0.77	10.38	2.67	2.14
HORVU3Hr1G036820	Peroxidase superfamily protein	15.71	9.60	0.47	23.64	2.89	2.73
HORVU3Hr1G036860	Peroxidase superfamily protein	51.93	11.88	0.31	62.45	7.93	9.29
HORVU3Hr1G036880	Peroxidase superfamily protein	26.65	11.70	0.68	43.24	5.97	3.99
HORVU3Hr1G074920	Peroxidase superfamily protein	13.86	1.40	0.00	13.78	0.90	0.41
HORVU3Hr1G074940	Peroxidase superfamily protein	1.28	1.09	0.54	19.53	1.98	1.26
HORVU3Hr1G074950	Peroxidase superfamily protein	6.22	1.82	0.14	19.50	1.28	1.21
HORVU3Hr1G074960	Peroxidase superfamily protein	22.31	16.86	11.43	144.88	29.09	24.82
HORVU3Hr1G077580	Peroxidase superfamily protein	12.31	10.34	3.67	78.00	16.38	7.80
HORVU3Hr1G079480	Peroxidase superfamily protein	10.78	5.34	0.13	60.77	2.49	0.55
HORVU3Hr1G091740	Peroxidase superfamily protein	1.51	1.39	0.00	17.06	0.78	0.55
HORVU4Hr1G022270	Peroxidase superfamily protein	1.68	1.32	0.46	10.69	1.71	1.36
HORVU4Hr1G022280	Peroxidase superfamily protein	0.96	0.84	0.12	4.90	0.78	0.13
HORVU4Hr1G050680	Peroxidase superfamily protein	14.13	3.91	0.14	15.53	2.63	0.65
HORVU4Hr1G065000	Peroxidase superfamily protein	3.30	3.95	3.17	33.19	7.95	6.76
HORVU5Hr1G043810	Peroxidase superfamily protein	2.04	0.49	0.00	2.46	0.18	0.00
HORVU5Hr1G046900	Peroxidase superfamily protein	25.90	8.46	1.77	86.83	9.73	6.06
HORVU5Hr1G070290	Peroxidase superfamily protein	2.47	1.99	0.78	8.04	1.31	0.26
HORVU5Hr1G097260	Peroxidase superfamily protein	7.77	5.17	1.14	9.70	3.71	1.53
HORVU5Hr1G097270	Peroxidase superfamily protein	29.16	8.77	9.52	55.26	11.19	6.05
HORVU6Hr1G010340	Peroxidase superfamily protein	3.59	0.45	0.00	4.88	0.75	0.30
HORVU6Hr1G020950	Peroxidase superfamily protein	5.23	1.87	2.07	6.16	0.72	0.70
HORVU6Hr1G075510	Peroxidase superfamily protein	22.87	6.06	0.23	26.60	3.62	3.30
HORVU6Hr1G087120	Peroxidase superfamily protein	3.36	1.25	0.03	5.23	0.81	0.17
HORVU7Hr1G011840	Peroxidase superfamily protein	330.08	73.44	7.40	137.88	53.48	14.70
HORVU7Hr1G037220	Peroxidase superfamily protein	11.48	5.45	0.19	8.97	1.76	0.68
HORVU7Hr1G054510	Peroxidase superfamily protein	6.60	1.86	0.03	7.04	0.65	1.11
HORVU7Hr1G080550	Peroxidase superfamily protein	12.65	4.99	0.23	75.88	7.08	0.40
HORVU7Hr1G089310	Peroxidase superfamily protein	5.73	0.28	0.00	2.52	0.39	0.20
HORVU7Hr1G089520	Peroxidase superfamily protein	1.31	0.18	0.00	3.49	0.00	0.00
HORVU7Hr1G091390	Peroxidase superfamily protein	23.07	2.19	0.10	18.21	1.98	0.22
HORVU7Hr1G093400	Peroxidase superfamily protein	5.79	0.70	0.21	7.10	0.40	0.02
HORVU7Hr1G098110	Peroxidase superfamily protein	5.41	1.51	0.10	8.92	1.43	0.30
HORVU7Hr1G098560	Peroxidase family protein	2.89	0.81	0.33	4.05	0.65	0.72
HORVU7Hr1G108210	Peroxidase superfamily protein	21.94	6.70	0.51	60.84	4.45	4.19
HORVU7Hr1G108220	Peroxidase superfamily protein	21.23	1.81	0.84	13.38	2.95	0.73
HORVU7Hr1G116550	Peroxidase superfamily protein	1.66	0.56	0.08	6.89	0.35	0.26
HORVU0Hr1G002770	Peroxidase superfamily protein	6.52	0.64	0.00	5.25	0.64	0.72
HORVU0Hr1G002800	Peroxidase superfamily protein	1.15	0.49	0.00	4.58	0.40	0.00
HORVU0Hr1G005850	Peroxidase superfamily protein	9.59	2.90	1.81	12.40	2.93	2.04
HORVU1Hr1G020800	Peroxidase superfamily protein	146.59	150.45	661.41	92.06	409.69	480.53
HORVU1Hr1G085790	Peroxidase superfamily protein	0.08	0.24	2.88	3.36	2.78	11.19
HORVU2Hr1G018370	Peroxidase superfamily protein	0.00	0.29	0.20	0.50	0.48	1.68
HORVU2Hr1G063460	Peroxidase superfamily protein	5.24	12.88	39.51	7.92	31.86	40.96
HORVU2Hr1G125200	Peroxidase superfamily protein	25.86	26.66	9.53	16.25	70.33	64.05
HORVU3Hr1G112040	Peroxidase superfamily protein	311.77	174.85	161.80	247.24	269.42	779.25
HORVU7Hr1G076120	Peroxidase superfamily protein	55.23	90.23	176.99	45.23	188.81	136.63
HORVU0Hr1G010070	Peroxidase superfamily protein	40.48	13.63	228.77	42.16	17.26	206.06
HORVU3Hr1G110310	ascorbate peroxidase 3	8.19	4.25	0.53	8.09	3.00	0.78
HORVU7Hr1G121700	catalase 2	110.01	82.77	534.36	131.17	189.35	883.12
HORVU2Hr1G096960	glutathione peroxidase 6	15.27	13.46	4.29	40.12	13.52	9.20
HORVU6Hr1G063830	glutathione peroxidase 6	37.43	24.64	8.18	112.65	30.15	21.95
HORVU5Hr1G057800	L-ascorbate oxidase	2.79	1.21	0.27	11.47	0.89	0.45
HORVU5Hr1G076430	L-ascorbate oxidase	7.76	3.78	0.13	66.08	3.95	0.90
HORVU5Hr1G076500	L-ascorbate oxidase	1.38	1.03	0.00	9.76	0.30	0.12
HORVU5Hr1G076510	L-ascorbate oxidase	6.18	2.28	0.13	40.66	2.12	0.35
HORVU7Hr1G087240	L-ascorbate oxidase	7.02	1.93	0.00	12.31	1.76	2.01
HORVU7Hr1G087250	L-ascorbate oxidase	0.31	0.60	0.52	3.37	1.99	0.38
**Cell wall Modifying enzymes**							
HORVU1Hr1G038500	Xyloglucan galactosyltransferase KATAMARI1 homolog	1.73	0.29	0.04	1.52	0.19	0.23
HORVU1Hr1G038510	Xyloglucan galactosyltransferase KATAMARI1 homolog	5.66	0.80	0.00	5.93	0.57	0.38
HORVU1Hr1G087320	xyloglucan endotransglucosylase/hydrolase 25	43.22	28.11	3.07	115.34	34.53	20.29
HORVU2Hr1G101160	xyloglucan endotransglucosylase/hydrolase 16	11.81	5.24	2.09	27.46	4.59	2.43
HORVU2Hr1G101240	xyloglucan endotransglucosylase/hydrolase 15	0.94	0.56	0.08	2.00	0.38	0.40
HORVU2Hr1G105610	Xyloglucan galactosyltransferase KATAMARI1 homolog	2.64	0.86	0.00	3.53	0.87	0.23
HORVU3Hr1G002770	Xyloglucan galactosyltransferase KATAMARI1 homolog	2.38	0.71	0.00	1.23	0.60	0.06
HORVU3Hr1G016820	xyloglucan endotransglucosylase/hydrolase 25	2.76	0.80	0.00	4.64	0.52	0.19
HORVU4Hr1G028720	xyloglucan endotransglucosylase/hydrolase 5	2.81	1.98	0.00	13.54	0.69	0.50
HORVU4Hr1G054910	xyloglucan xylosyltransferase 5	19.67	12.28	1.89	86.45	17.00	11.99
HORVU4Hr1G064220	xyloglucan endotransglucosylase/hydrolase 28	8.03	8.44	2.05	31.66	7.31	5.28
HORVU4Hr1G078990	Xyloglucan galactosyltransferase KATAMARI1 homolog	6.11	1.31	0.03	5.14	1.30	0.35
HORVU4Hr1G079010	Xyloglucan galactosyltransferase KATAMARI1	4.91	0.77	0.00	5.01	0.53	0.59
HORVU4Hr1G079020	Xyloglucan galactosyltransferase KATAMARI1 homolog	2.29	0.49	0.00	2.24	0.34	0.05
HORVU5Hr1G042000	xyloglucan xylosyltransferase 5	7.80	2.77	0.12	15.60	1.84	0.36
HORVU7Hr1G012600	xyloglucan endotransglucosylase/hydrolase 25	16.48	3.88	0.46	38.23	2.81	3.36
HORVU7Hr1G081740	xyloglucan endotransglucosylase/hydrolase 26	0.89	0.40	0.16	4.70	0.41	0.33
HORVU7Hr1G086890	xyloglucan endotransglucosylase/hydrolase 32	2.47	0.45	0.00	5.57	0.39	0.03
HORVU7Hr1G106530	xyloglucan endotransglucosylase/hydrolase 16	11.01	3.94	0.28	13.90	1.44	0.21
HORVU0Hr1G021280	xyloglucan endotransglucosylase/hydrolase 28	0.49	1.55	0.24	4.61	0.33	0.98
HORVU2Hr1G101150	xyloglucan endotransglucosylase/hydrolase 13	17.43	18.28	82.69	13.96	38.05	176.00
HORVU7Hr1G021820	xyloglucan endotransglucosylase/hydrolase 25	0.54	0.50	2.00	0.50	1.39	2.41
HORVU7Hr1G021950	xyloglucan endotransglucosylase/hydrolase 25	1.08	0.85	2.60	1.55	2.41	5.68
HORVU7Hr1G098370	Xyloglucan endotransglucosylase/hydrolase family protein	1.34	6.04	57.29	1.66	5.22	40.90
HORVU5Hr1G014500	Pectinesterase inhibitor domain containing protein	1.01	0.11	0.00	0.96	0.34	0.00
HORVU2Hr1G032220	pectinesterase 11	0.27	0.19	4.06	0.92	0.42	7.85
HORVU3Hr1G056440	pectinesterase 11	0.00	0.11	3.16	0.13	0.06	7.59
HORVU0Hr1G013380	respiratory burst oxidase homologue D	0.80	0.65	0.32	6.10	0.92	0.28
HORVU1Hr1G072140	respiratory burst oxidase homologue D	0.34	0.20	0.01	2.56	0.26	0.18
HORVU1Hr1G072160	respiratory burst oxidase homologue D	0.79	0.60	0.05	5.09	0.34	0.26
HORVU4Hr1G081670	respiratory burst oxidase homologue D	26.26	26.41	168.24	26.46	177.33	160.69
HORVU4Hr1G086500	respiratory burst oxidase homolog B	16.61	4.28	0.53	20.66	7.75	2.26
HORVU5Hr1G024550	respiratory burst oxidase homologue D	1.96	19.64	17.91	11.58	47.65	19.41
HORVU5Hr1G078630	respiratory burst oxidase homologue D	6.22	4.98	1.40	52.28	8.07	3.11

Hormones play an important role in the plant response to environmental stress. Here, we identified some DEGs related to hormones that are involved mainly in the biosynthesis of ethylene and auxin. Ethylene is biosynthesized by the activation of 1-aminocyclopropane-1-carboxylicacid synthase (ACS) and ACC oxidase (ACO). Two ACSs and 6 ACOs were identified in TX9425 and Franklin. Two ACO genes (*HORVU5Hr1G067490* and *HORVU5Hr1G067530*) were significantly accumulated in both varieties, but the genes inductions in TX9425 were greater. Thirty-one DEGs involved in auxin metabolism were identified in the two genotypes, including 23 downregulated and 8 upregulated genes. After 72 h of waterlogging treatment, the expression levels of *HORVU1Hr1G025670*, *HORVU3Hr1G064590* and *HORVU3Hr1G084840* in TX9425 were significantly higher than the levels in Franklin (Table 3).

Reactive oxygen species (ROS), which are produced when plants experience adverse stresses, can damage normal functions in plant cells. To survive, plants have evolved multiple strategies such as activating antioxidant systems to remove excess ROS. A total of 124 DEGs involved in ROS scavenging were found in our study, and most of them were downregulated. These DEGs are involved in the synthesis of glutathione S-transferase, peroxidase, catalase, and L-ascorbate oxidase, most of which (82 genes, 66.12% of 124) were related to peroxidase. Eight genes related to glutathione S-transferase and 8 genes related to peroxidase were upregulated in both genotypes, and the fold changes of these genes in TX9425 were significantly higher than the fold changes in Franklin (Table 3).

To adapt to waterlogging stress, plants also have evolved many mechanisms, such as the formation of adventitious roots and aerenchyma. The formation of aerenchyma was related to cell wall biosynthesis and loosening. As expected, we found that 34 DEGs were involved in cell wall modifying enzymes, such as xyloglucan galactosyltransferase, pectinesterase, and respiratory burst oxidase homologue. Eight DEGs were significantly upregulated in both genotypes. Under waterlogging stress, the genes *HORVU2Hr1G101150* and *HORVU4Hr1G081670* in TX9425 had significantly higher expression levels than those genes in Franklin (Table 3).

### Overexpression of HvADH4 enhanced waterlogging tolerance by the increasing ROS scavenging capacity

A total of 44 *ADH* genes were identified in the barley genome based on the BLAST program. These genes were named *HvADH1*- *HvADH44* according to their order of distribution on the chromosomes (Table S4). In the *HvADH* gene family, the length of coding sequences ranged from 99 bp (*HvADH17*) to 1524 bp (*HvADH37*). The size of the corresponding amino acids varied between 32 and 507.The theoretical isoelectric point (PI) of these genes ranged from 4.51 to 9.66, and the molecular weight (Mw) varied from 3.47 to 48.11 kDa.

**Fig. 6:**
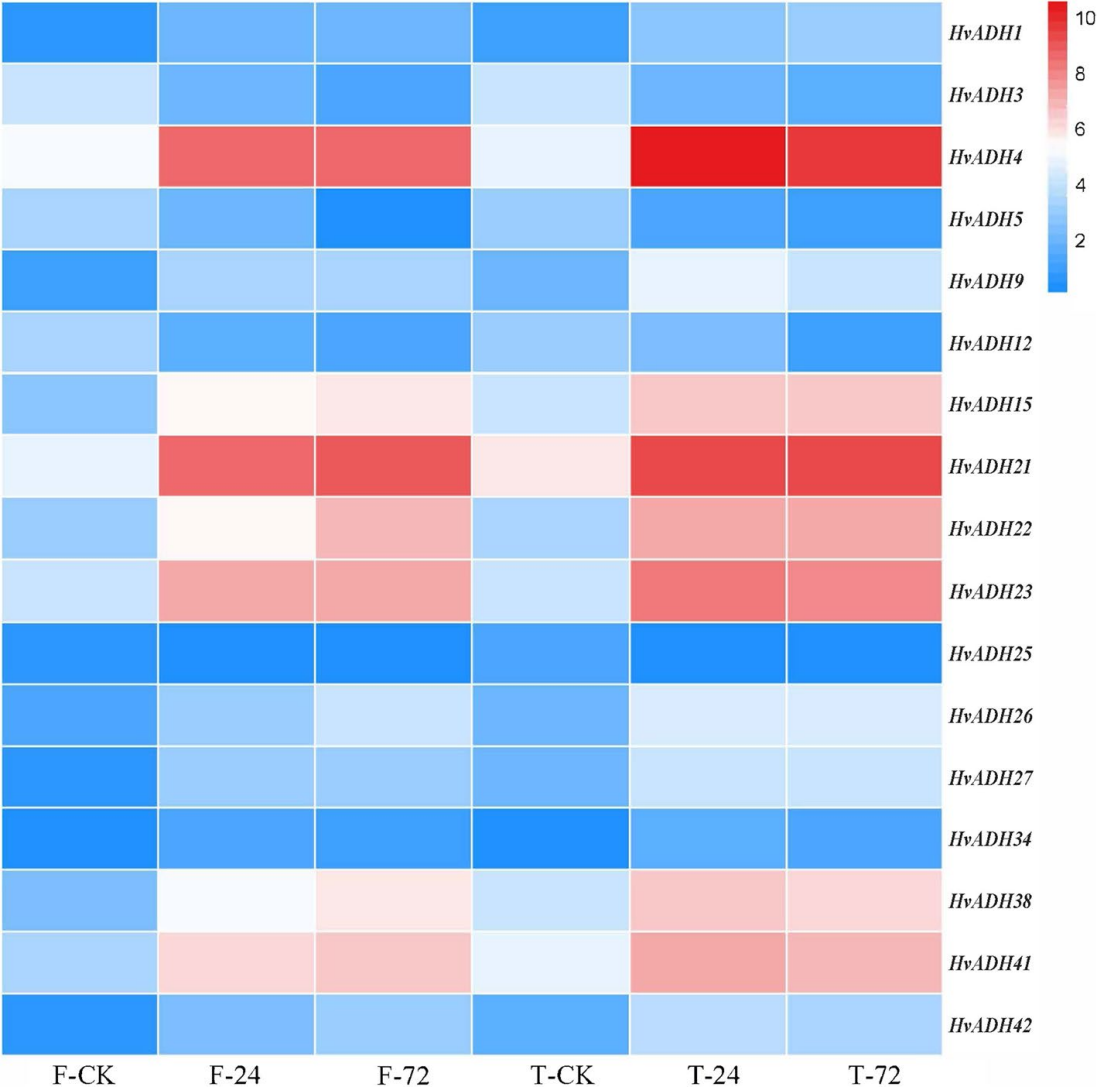
The expression difference levels of DEGs involved in alcohol dehydrogenase between waterlogging treatment and control in TX9425 and Franklin

**Fig. 7:**
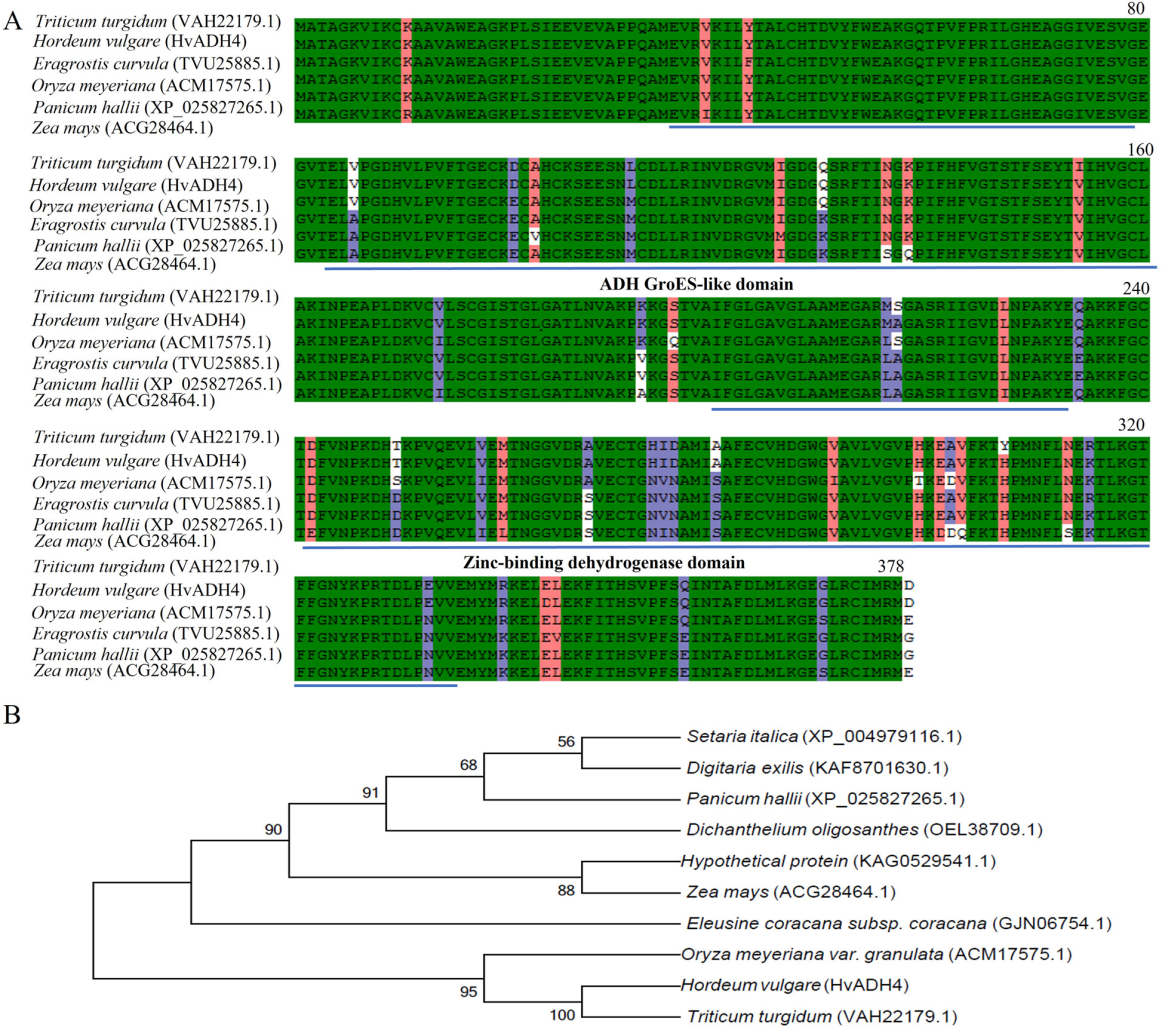
Amino-acid sequence alignment and phylogenetic tree analysis of ADHs from barley and other plant species. (**A**) Conserved domain alignment of ADHs from different plant species by Clustal W. (**B**) Phylogenetic tree analysis of ADHs from different plant species by MEGA 6.0 with Neighbor-Joining method

In this study, 17 *ADH* genes were found to have differential expression between the waterlogging treatment and the control, except for *HvADH25* in Franklin (Fig. 6). The highest differential expression was found for *HvADH4* in TX9425, and there was an approximately 50- fold difference between 24 h and the control. We thus performed a standard method to isolate *HvADH4* from TX9425. Sequencing of *HvADH4* showed that the full-length gene was 1158 bp in length and encoded 385 amino acids. Multiple amino acid alignment showed that the *HvADH4* protein shared two highly conserved ADH GroES-like (amino acids 36–156) and zinc-binding dehydrogenase domains (amino acids 205–336) (Fig. 7A). The phylogenetic tree indicated that *HvADH4* has relatively high homology with proteins from *Triticum turgidum*, and relatively distant sequence homology with the proteins from *Setaria italica* (Fig. 7B).

**Fig. 8:**
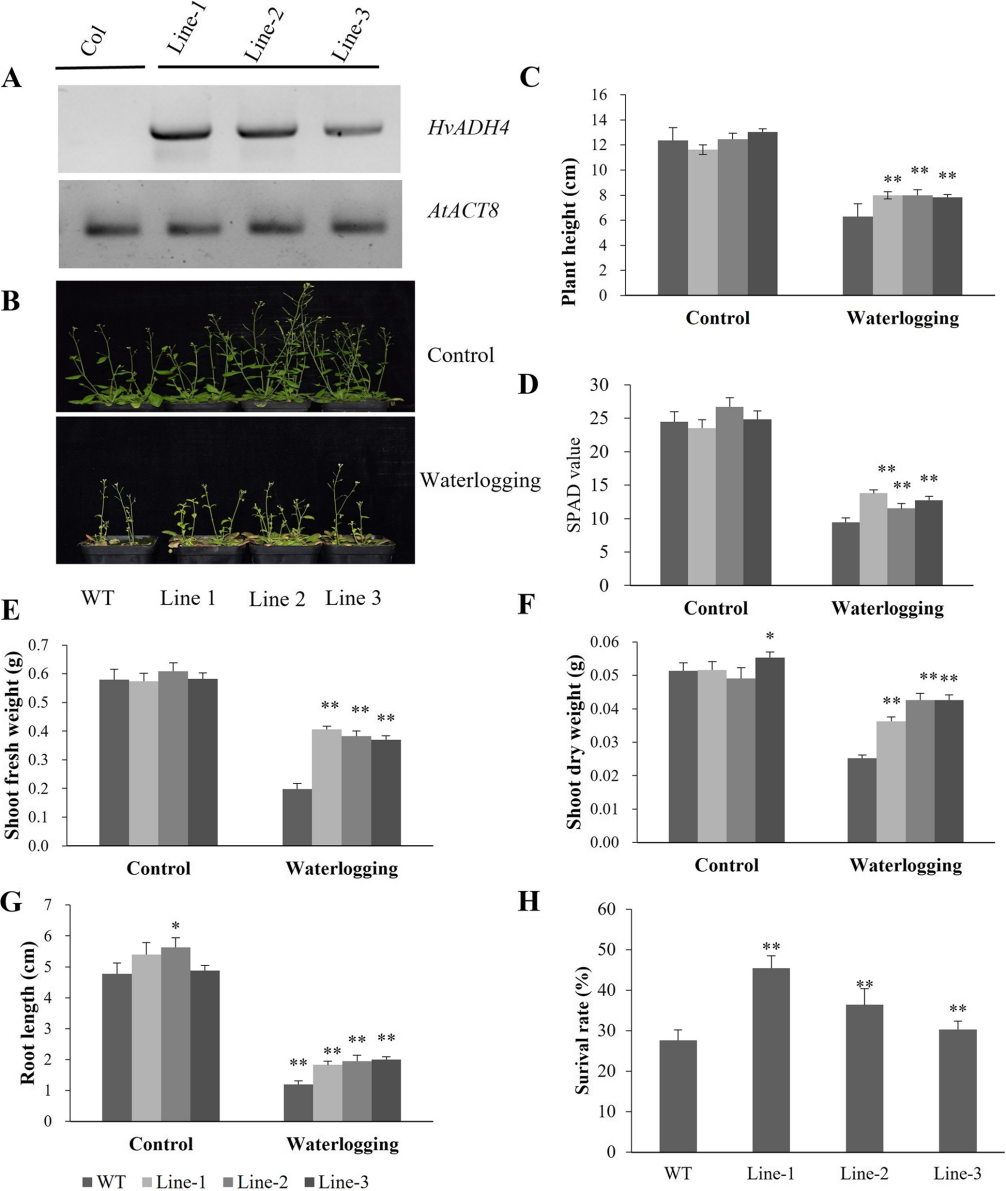
Waterlogging tolerance assay of *HvADH4* overexpression lines (Line1, Line2, Line 3) and wild-type (WT). (**A**) RT-PCR analysis of transgenic and wild-type plants. *AtACT8* was chosen as control gene. (**B**) Five-week-old plants were subjected to waterlogging stress for further 2 weeks. (**C**) Plant height. (**D**) Soil–plant analysis development (SPAD) value (based on chlorophyll meter reading). (**E**) Shoot fresh weight. (**F**) Shoot dry weight. (**G**) Root length. (**H**) Surival rate in the wild-type and *HvADH4* transgenic lines were measured under control and waterlogging stress. Values are the means ± SD. Means were generated from three independent measurements. Asterisks indicate significant differences between transgenic plants and WT according to Student’s t-test (* *p* < *0.05*; ** *p* < *0.01*)

To further verify the function of barley *HvADH4* (*HORVU1Hr1G082250*), transgenic *Arabidopsis* plants overexpressing the *HvADH4* gene from TX9425 were generated. Five-week-old plants of the WT and three homozygous T3 transgenic lines were selected for waterlogging stress experiments. Transgenic plants that expressed *HvADH4* were confirmed by RT-PCR (Fig. 8A). Under normal growth conditions, the transgenic Line-2 and Line-3 grew better than the WT, while the differences were found to be statistically insignificant (Fig. 8B). Under waterlogging conditions, plant height was reduced by 49.1% in the WT, and 31.2, 36.1 and 40.3% in the transgenic lines (Fig. 8C). Compared to the control, the SPAD value was 61.6% lower in the WT, and 41.4, 51.4, 48.8% lower in the transgenic lines (Fig. 8D). The shoot fresh weights of the transgenic lines were 29.2, 37.2 and 36.5%, respectively, which were lower than those weights in the control, and 65.8% smaller than those weights in the WT (Fig. 8E). The shoot dry weight decreased by 51.0% in the WT, and by 29.7, 13.3 and 22.9% in the transgenic lines (Fig. 8F). In addition, the root lengths of the WT plants decreased more than the root lengths of the transgenic lines during waterlogging stress (Fig. 8G). Furthermore, the average survival rate of the transgenic lines after waterlogging was 81.8%, but the average survival rate of the WT was only 37.4% (Fig. 8H). Taken together, these data indicate that the overexpression of *HvADH4* in *Arabidopsis* significantly enhances plant waterlogging tolerance.

**Fig. 9:**
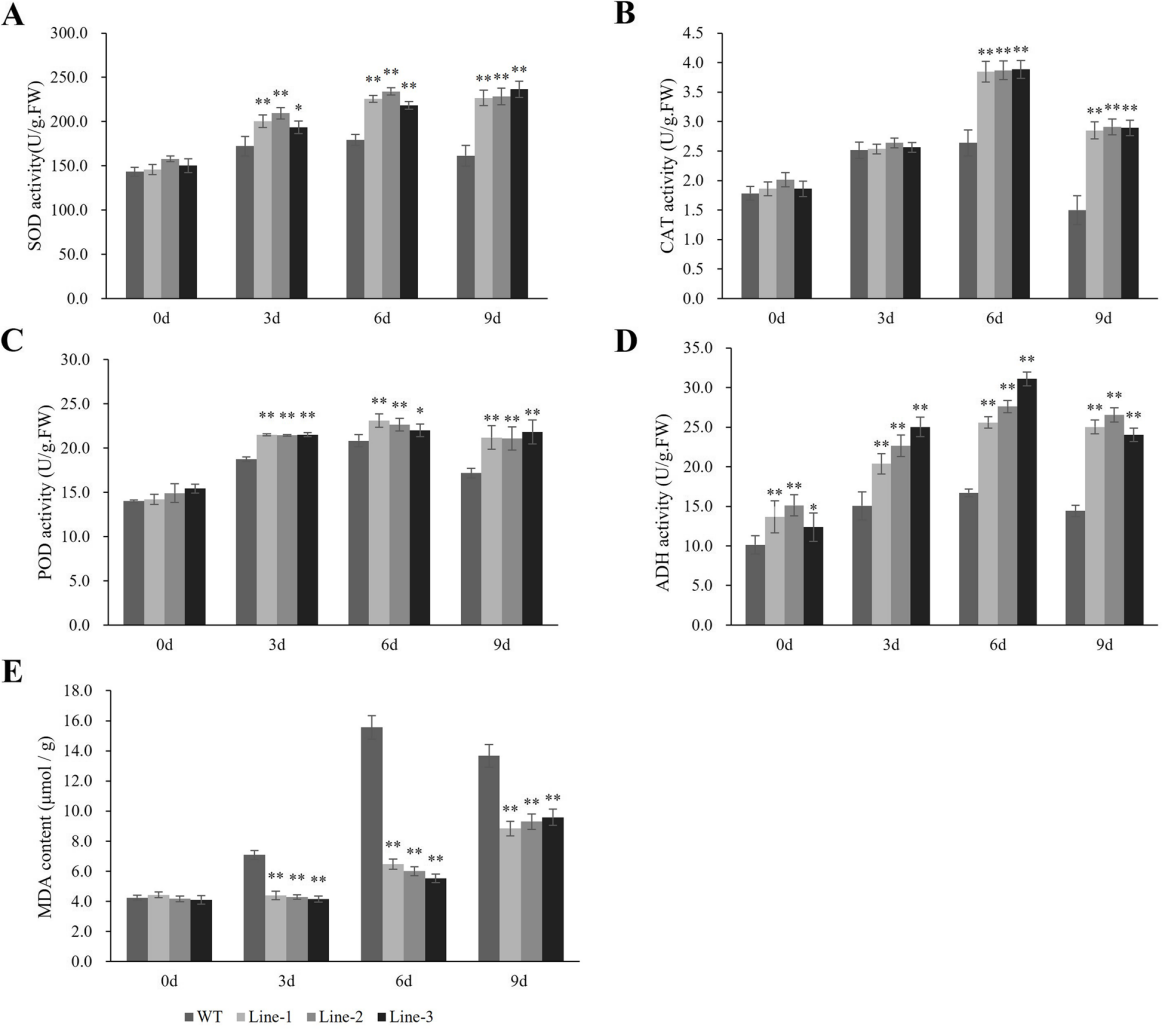
Analysis of SOD, CAT, POD, ADH activities and proline content were carried out in transgenic lines and WT under waterlogging stressed conditions. SOD, CAT, POD, ADH and proline levels. (**A**–**E**) were measured in the leaves of plants subjected to waterlogging stress 3 days, 6 days, 9 days. The mean value and standard deviation were obtained from three independent experiments. The data represent mean ± SD of three biological repeats with three measurements per sample. Asterisks indicate significant differences from WT as determined using Student’s t-test (* *p* < *0.05*; ** *p* < *0.01*)

To investigate the difference in the physiological response to waterlogging stress between the WT plants and the transgenic plants, the activities of antioxidant enzymes (SOD, CAT, and POD), ADH activity and MDA content were examined under normal and waterlogging conditions. The transgenic lines showed higher ADH activity than the WT plants even when they were under control conditions, and ADH activity remained significantly higher at subsequent times (Fig. 9D). There were no significant differences in the activity of antioxidant enzymes between transgenic lines and WT under normal growth conditions. After waterlogging, the major antioxidant enzyme activity increased markedly in both WT and transgenic plants, reaching peak levels at 6 days of treatment and then decreasing after 9 days of treatment. However, the fold changes were significantly greater in the transgenic lines than in the WT (Fig. 9 A, B, C). MDA content is an important indicator to measure the level of lipid peroxidation. As shown in Fig. 9E, the MDA content in WT plants was significantly higher than the MDA content in transgenic lines, and this difference was more pronounced in the 6 d samples. Therefore, these results suggest that the overexpression of *HvADH4* enhanced the scavenging ability of ROS in the plants and reduced the oxidative damage of plants under waterlogging stress.

## Discussion

### Morpho-anatomical responses to waterlogging stress in barley

Waterlogging tolerance is a complicated trait, both physiologically and genetically [26, 27]. Waterlogging-induced plant growth inhibition is pervasive, including decreased dry weight (DW) in shoots/roots, leaf area, plant height, and chlorophyll content, and this inhibition also causes yield penalty [4]. Franklin was severely affected after 21 days of waterlogging treatment compared with TX9425, including plant height, tiller number, leaf area, shoot fresh weight and dry weight. Franklin shoots appeared wilted and presented more yellow leaves under waterlogging than the control. Remarkably, there are more morphological adaptations in waterlogging-tolerant TX9425 than the susceptible Franklin.

The root is the first organ affected by waterlogging stress. The formation of new adventitious roots and aerenchyma is one of the most important characteristics that adapts to hypoxic environments [5, 28]. Newly formed adventitious roots contain more aerenchyma, which not only provides a gas diffusion space with increased O_2_ transport from shoots to roots but also reduces the number of oxygen-consuming cells [29, 30]. In the present study, TX9425 had significantly more adventitious roots and aerenchyma than Franklin under waterlogging stress. This phenomenon has been reported in cucumber [23] and maize [31].

### Waterlogging affects energy metabolism in barley

The energy metabolism pathway is critical for plant survival under low-oxygen stress and is related to starch and sucrose metabolism and glycolysis fermentation. As expected, we found that some DEGs, such as sucrose synthase 4, pyruvate kinase family protein, ATP-dependent 6-phosphofructokinase, fructose-bisphosphate aldolase 2, alcohol dehydrogenase, and pyruvate decarboxylase-2, were all upregulated after waterlogging. Compared to Franklin, the transcription levels of these genes were higher in TX9425 under both control and waterlogging conditions (Table 3).

Sucrose synthase (SUS) and sucrose phosphate synthase are key enzymes for the hydrolysis of sucrose, which play crucial roles in providing adequate sugar supply under waterlogging stress [32]. In low oxygen environments, the genes related to sucrose synthase in *Arabidopsis* [33], cucumber [23], and *P. arundinacea* [34], were all significantly upregulated. In addition, knockout of *SUS1* and *SUS4* induced less tolerance to oxygen deficits compared to wild-type in maize [35] and *Arabidopsis* [36]. The overexpression of sucrose synthase genes in cucumber confers tolerance against hypoxia stress [37]. Here, the expression of sucrose synthase 4 and sucrose phosphate synthase 1F were higher in TX9425 than in Franklin under 24 h of waterlogging stress. Pyruvate decarboxylase (PDC) is one of the key enzymes of ethanolic fermentation. *PDC1* and *PDC2* play an important role in waterlogging stress tolerance in *Arabidopsis* and *Actinidia deliciosa* [38]. In this study, only *PDC2* was significantly upregulated under waterlogging stress (Table 3). This result suggests that TX9425 can accumulate more energy by decomposing more carbohydrates and amino acids, making it more readily adaptable to hypoxia stress.

### Responses of ROS and hormones to waterlogging

Ethylene is an important hormone in response to waterlogging stress in plants, and can facilitate adventitious root and aerenchyma formation [5, 39]. In our previous study, we found that the ethylene content distinctly increased in TX9425 after waterlogging stress [40]. The RNA-Seq analysis showed that the expression of two ACO (*HORVU5Hr1G067490* and *HORVU5Hr1G067530*) accumulated in both lines, while the gene expression in TX9425 was much greater. The results were consistent with published proteomic studies [41]. Auxin regulates the development of the lateral roots and plays a role in root growth [42]. Thirty-one genes involved in auxin metabolism were identified in this research, and most of them were downregulated. Interestingly, auxin storage was negatively associated with adventitious root initiation in cucumber [23].

Hypoxia stress can cause plants to overproduce ROS, which can cause progressive oxidative damage. To respond to oxidative stress plants have developed antioxidant defence systems, including SOD, CAT, POD, and GST [43]. Wang et al. [44] observed that the activities of antioxidant enzyme activity increased in soybean under waterlogging conditions. In contrast, Wang et al. [34] found that the activities of SOD, CAT, and POD were significantly decreased in *Phalaris arundinacea* in response to waterlogging stress. The reason for these two different results may be due to different treatment times and genotypes [45]. In this study, we identified 124 DEGs associated with the antioxidant system, in which most genes were downregulated. Similar to POD enzyme activity, 8 genes related to POD were upregulated in both genotypes, and the fold changes of these genes in TX9425 were significantly higher than in Franklin. In addition, one CAT gene (*CAT2*) in both lines was also upregulated after waterlogging treatment, indicating that this gene is a key regulator of CAT enzyme activity.

Xyloglucan endotransglycosylase/hydrolase (XTH) enzymes play a role in the loosening of cell walls and affect cell proliferation. XTH enzymes are involved in plant growth and resistance to stress [46]. We found that 24 DEGs were involved in XTH, and *XTH 13* was significantly upregulated in TX9425 under waterlogging stress (Table 3). The present study suggests that *XTH 13* plays an important role in waterlogging tolerance of barley.

### Importance of the ADH gene in the response to waterlogging stress of barley

ADH is a major fermentative enzyme for oxidizing ethanol to acetaldehyde, which play a key role in resistance to waterlogging [21]. *ADH* family genes from tomato [47], rice [48], *Pyrus bretschneideri* [49] and wheat [50] have been detected at the whole genome level. Twenty-two *ADH* genes have been identified in the wheat genome database [50], and we identified 44 *ADH* genes in the barley genome.

The expression of *ADH* genes has been observed to be significantly elevated in soybean roots under hypoxia stress [51]. Shen et al. [50] found that *TaADH1/2*, *TaADH3* and *TaADH9* play an important role in the waterlogging tolerance of wheat, which was significantly induced by waterlogging. To further validate *ADH* gene function, some transgenic assays were conducted. Overexpression of *ADH* genes of soybean and kiwifruit increased waterlogging tolerance in transgenic plants [20, 21]. In contrast, overexpression of the *Arabidopsis ADH1* gene and increased ADH activity do not affect ethanol levels and flooding survival tolerance under hypoxic conditions compared to wild-type cells [19]. Thus, the function of *ADH* genes varies with the different plants and stages. In this study, 17 *ADH* genes were differentially expressed, of which 7 genes were significantly upregulated after waterlogging stress. *HvADH4* of TX9425 showed the highest level of differential expression. Overexpression of *HvADH4* in transgenic *Arabidopsis* enhanced plant waterlogging tolerance, which could be caused by increased activity of fermentation and antioxidant enzymes.

Waterlogging is a complex trait controlled by numerous QTLs. So far, many QTLs associated with waterlogging tolerance have been successfully mapped using bi-parental linkage mapping based on various waterlogging related traits [7, 52–55]. The results of QTL mapping largely depend upon the two parents, population size, type of markers, and density of markers, and so on. For example, it has been reported that two major QTLs were mapped on 2H and 4H [52, 54]. However, Broughton et al. found that 10 QTLs associated with waterlogging-tolerant were mapped on 1H [7]. Cloning these genes have not been reported up to now. RNA-Seq mainly used to analyze gene expression with high-throughput sequencing. *HvADH4* might be one of the downstream target gene under waterlogging stress. Candidate genes related to waterlogging stress in barley will be analyzed by GWAS and QTL.

In the present study, only wild-type *Arabidopsis* was used as a control, and transgenic line contains empty vector transgene were not used. Thus, the waterlogging tolerance of transgenic *Arabidopsis* lines might be attributed to the insertion of vectors in the *Arabidopsis* genome rather than the overexpression of *HvADH4* gene. In addition, ectopic overexpression of a gene might not reflect its intrinsic function. In the future, the functions of the related genes will be further verified by over-expression, RNAi and gene editing in barley.

## Conclusions

In this study, two barley varieties with different waterlogging tolerances were subjected to waterlogging treatment. Analyses of the morphological and physiological indicators revealed that TX9425 exhibited stronger waterlogging tolerance than Franklin. To further explore the mechanism controlling waterlogging tolerance in barley, RNA-seq analysis was performed. We identified 3064, 5693 and 2297, 8462 DEGs in TX9425 at 24 h, 72 h and Franklin at 24 h, 72 h, respectively. GO and KEGG analyses showed that the waterlogging tolerance of barley was closely related to energy metabolism, hormone regulation, ROS scavenging, and cell wall-modifying enzymes. Seventeen *ADH* genes were identified to be waterlogging responsive in barley. Among these genes, the expression level of *HvADH4* was significantly different between the control and waterlogging groups. In addition, transgenic *Arabidopsis* with *HvADH4* has improved waterlogging tolerance by deterring ROS accumulation. This work demonstrated that the *HvADH4* gene plays an important role in waterlogging stress response.

## Methods

### Plant materials and treatments

TX9425 is waterlogging-tolerant feed barley originating from China, while Franklin is waterlogging-sensitive Australian malting barley [56]. The two accessions were obtained from the National Crop Genebank of China (NCGC, Beijing). The seeds of two genotypes were sown in plastic pots (22 cm × 25 cm) filled with the mixture of nutritional substance and vermiculite. Plants were grown in a greenhouse at a temperature of 20 °C/day and 15 °C/night. Waterlogging treatments started at the four-leaf stage and lasted for three weeks. The waterlogged plants were irrigated with tap water to 2–3 cm above the nutritional substance surface. The control plants were irrigated as needed to avoid drought stress or waterlogging stress. The experiment was carried out with three biological replications. After 21-day treatment, roots and leaves were collected and carefully washed with water for further analysis.

### Morphological characteristic investigation

The leaf area and physiological traits of leaf were measured on the first fully expanded leaf below the shoot apex. Leaf chlorosis is the proportion of each plant that loses its green color (yellow) [56].

The roots were carefully rinsed with hydropneumatic elutriation device and detached from their nodal bases. Adventitious roots were arranged and floated on shallow water in a glass tray (30 cm × 30 cm), then scanned using Epson Expression 1680 scanner (Seiko Epson Corp, Japan), finally analyzed using WinRHIZO Root Analyzer System (Regent Instruments Inc., Canada) to measure length, diameter, surface area and root volume [57]. The parameters related to adventitious roots were measured with three biological replications, and six plants were selected for each replicate.

### Tissue anatomy

After 21-day waterlogging treatment, samples were obtained from the mature zone of adventitious root (approximately 6 cm from the root apex), shoot base (root node), and leaf (the first fully expanded leaf below the shoot apex). They were cut into 0.5 cm segments, and immediately immersed in 0.1 M glutaraldehyde-phosphate buffer fixative (pH 7.2) at 4 °C for at least 3 h. Subsequently, the samples were dehydrated in a graded ethanol series of 20%, 40%, 60%, 80%, 90%, 95%, and 100% (15 min each). Ethanol was replaced with propyleneoxide, and the tissues were infiltrated and embedded in SPI low-viscosity Spurr’s resin [58]. Sections of 1 μm thick were cut with a glass knife on a Leica Ultracut R (Leica Microsystems, Inc., Germany), stained with 0.5% methyl violet for 10 min, and photographed under a light microscope (Leica, Germany). Root aerenchyma area and total root cross-sectional area were measured using the Image-pro plus (IPP) software (Media Cybernetics, USA).

### Physiological trait analysis

Fresh leaves and roots (0.5 g each) were rinsed thoroughly with distilled water. The crude enzymatic extracts of each line were prepared in 0.05 M phosphate buffer (pH 7.8) after grinding with a pestle and milling to powder in liquid nitrogen. The homogenate was filtered through four layers of muslin cloth and centrifuged at 12 000 g for 10 min at 4 °C. The final supernatants were used for physiological and biochemical assays. Chlorophyll content was determined by using the SPAD-502. The activities of superoxide dismutase (SOD), peroxidase (POD), catalase (CAT) and the content of malondialdehyde (MDA) and alcohol dehydrogenase (ADH) were measured using the corresponding assay kits (Institute of Jiancheng Bioengineering, Nanjing, China) according to the manufacturer’s instructions [40].

### RNA-seq and Transcription analysis

The root of TX9425 and Franklin were collected after waterlogging treatment for 24 h, 72 h, and control without waterlogging. Each treatment was processed with three biological replicates. Total RNA was extracted using the Plant RNA Purification Kit (Tiangen, Beijing, China). Twelve RNA-seq libraries (two accessions × two treatment × three biological replicates) were constructed by Novogene Bioinformatics Technology (Beijing, China) and sequenced by an Illumina HiSeq 2500 platform. Detailed process of transcriptome analysis as described in previous research [59]. The sequencing data were deposited in the NCBI SRA database (Bioproject ID: PRJNA889532). DEGseq was used to identify differentially expressed genes for RNA-seq data between waterlogging treatment and control. And the DEGs were further filtered with *P* value ≤ 0.05 and log_2_ fold change (log_2_ FC) ≥ 1.

### Quantitative real-time RT-PCR

To confirm the reality of candidate genes screened from RNA-seq. 10 candidate genes were selected to further validate by quantitative (qRT-PCR). The method of qRT-PCR was described as previous report [40]. The specific primers used for target were designed using the Primer 6. All the primers are listed in Supplementary Table S5. The *Hvactin*, *AtACT8* genes were used as the internal control. Target genes’ relative expression levels were determined as 2^−△△Ct^. Three biological replicates and three technical repeats were performed in all the qRT-PCR experiments.

### Cloning and bioinformatic analysis of HvADH4

Total RNA was extracted from barley leaves of TX9425, and cDNA was synthesized as a template by M-MLV reverse transcriptase (TaKaRa, Otsu, Shiga, Japan) according to the manufacturer’s instructions. Primers of *HvADH4* full-length CDS were designed by Primer 6. The PCR products were detected using agarose gel electrophoresis (1.0%). The sequence accuracy of the cloned genes was confirmed by DNA sequencing. The amino acid composition was analyzed with DNAMAN 9.0 software. The molecular weight and pI were examined the online software of ExPASy ProtParam (http://web.expasy.org/protparam/). Homologs of *HvADH4* in other plant species were analyzed by the BLAST tool of NCBI (https://blast.ncbi.nlm.nih.gov/Blast.cgi). MEGA 7.0 program was used for phylogenetic tree mapping by the neighbor-joining method and 1000 bootstrap replicates.

### Candidate gene validation by transgenic Arabidopsis

To further verify the candidate gene, transgenic *Arabidopsis* plants were generated by floral dipping. The detailed design and methods have been previously described [52]. The Gateway technology (Invitrogen, USA) was used to constructed transgenic lines. Through the floral dipping method, recombinant vectors were transferred into *Arabidopsis* (Columbia) using the *Agrobacterium tumefaciens* strain GV3101 (Clough and Bent,1998). The transgenic lines were selected by germinating the seeds in a MS medium containing 30 mg/L hygromycin. 40 resistant seedlings (T1 generation) were transplanted to the greenhouse after two weeks. T2 transgenic plants with a 3:1 (resistant: nonresistant) segregation ratio were selected. Seeds of 6 homozygous plants from T2 lines were screened (T3 generation) for 100% resistance. Further genetic analysis was performed using the homozygous T3 generation. Five-week-old *Arabidopsis* plants (T3 lines) were used for waterlogging treatment. The control plants were kept in normal conditions with regular watering. After the treatment of two weeks, the phenotypic and physiological traits were observed and recorded.

### Data analysis

For phenotypic, physiological parameter, and gene expression analysis was analyzed by Student’s t-test through the SPSS software. *and** represent the significant differences at *p* < *0.05* and *p* < *0.01*, respectively. All data were presented as mean ± standard deviation (SD) and were measured at least three times. And three biological replicates were set.

## Supplementary Information

**Additional file 1:** **Table S1.** The information of transcriptome libraries.

**Additional file 2:** **Table S2.** Summary of GO results of the differentially expressed genes.

**Additional file 3:** **Table S3.** KEGG enrichment analysis of the differentially expressed genes.

**Additional file 4:** **Table S4.** Properties and locations of the predicted HvADH proteins in barley.

**Additional file 5:** **Table S5.** qRT-PCR and cloning primers used in this study.

**Additional file 6.** 

## Data Availability

The transcriptome datasets supporting the conclusions of this study are available in the NCBI (BioProject: PRJNA889532).
