# Supplementary figures and images for: Transcriptome analysis of barley (Hordeum vulgare L.) under waterlogging stress, and overexpression of the HvADH4 gene confers waterlogging tolerance in transgenic Arabidopsis
Source: BMC Plant Biol. 2023 Jan 30;23:62. doi: 10.1186/s12870-023-04081-6 (PMC9885653; doi:10.1186/s12870-023-04081-6)

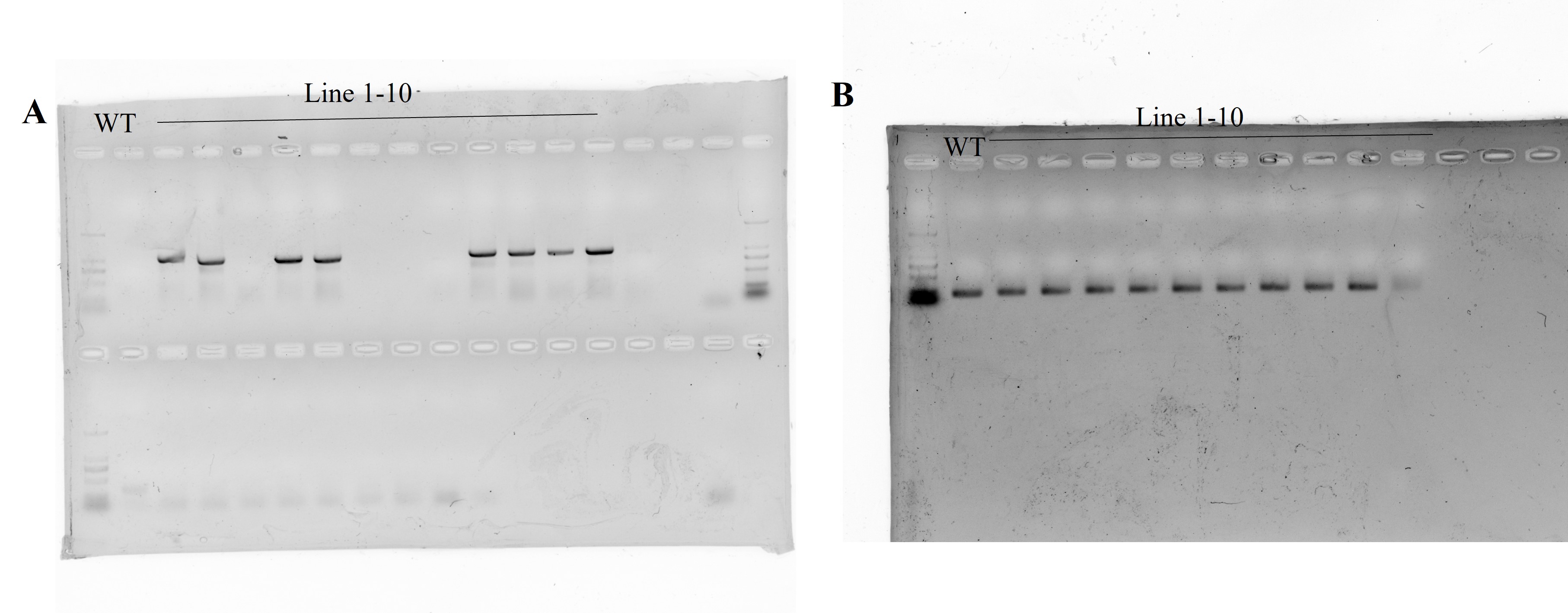

Supplement: Supplementary file 6 — Additional file 6. [file 12870_2023_4081_MOESM6_ESM.jpg]
